# What is the *real* value of omics data? Enhancing research outcomes and securing long-term data excellence

**DOI:** 10.1093/nar/gkae901

**Published:** 2024-10-17

**Authors:** Eva Price, Felix Feyertag, Thomas Evans, James Miskin, Kyriacos Mitrophanous, Duygu Dikicioglu

**Affiliations:** Department of Biochemical Engineering, University College London, Gower Street, London WC1E 6BT, UK; Oxford Biomedica (UK) Ltd, Windrush Court, Transport Way, Oxford OX4 6LT, UK; Oxford Biomedica (UK) Ltd, Windrush Court, Transport Way, Oxford OX4 6LT, UK; Oxford Biomedica (UK) Ltd, Windrush Court, Transport Way, Oxford OX4 6LT, UK; Oxford Biomedica (UK) Ltd, Windrush Court, Transport Way, Oxford OX4 6LT, UK; Department of Biochemical Engineering, University College London, Gower Street, London WC1E 6BT, UK

## Abstract

A wealth of high-throughput biological data, of which omics constitute a significant fraction, has been made publicly available in repositories over the past decades. These data come in various formats and cover a range of species and research areas providing insights into the complexities of biological systems; the public repositories hosting these data serve as multifaceted resources. The potentially greater value of these data lies in their secondary utilization as the deployment of data science and artificial intelligence in biology advances. Here, we critically evaluate challenges in secondary data use, focusing on omics data of human embryonic kidney cell lines available in public repositories. The emerging issues are obstacles faced by secondary data users across diverse domains as they concern platforms and repositories, which accept deposition of data irrespective of their species type. The evolving landscape of data-driven research in biology prompts re-evaluation of open access data curation and submission procedures to ensure that these challenges do not impede novel research opportunities through data exploitation. This paper aims to draw attention to widespread issues with data reporting and encourages data owners to meticulously curate submissions to maximize not only their immediate research impact but also the long-term legacy of datasets.

## Graphical Abstract

**Graphical Abstract:**
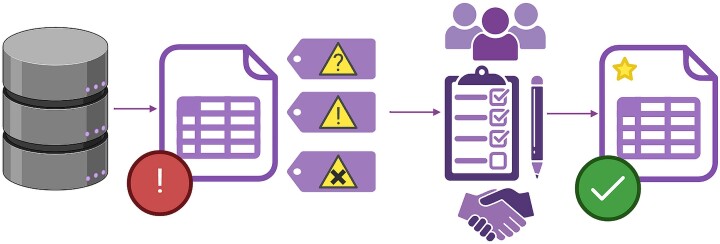

## Introduction

Secondary data use is pivotal in scientific research, unlocking insights and maximizing the value of pre-existing data. Researchers typically repurpose data to address new questions or re-evaluate findings reported using different experimental approaches or methods (1). Researchers can enhance the value of both their own work and existing data by integrating insights from high-throughput data into their research. This strategy increases the overall utility of the data, making it valuable for reuse across various fields. Centralized omics data repositories facilitate effective data reuse, fostering collaboration and accelerating discoveries.

Secondary data usage has had revolutionary impacts in the scientific world, as exemplified by several groundbreaking achievements. AlphaFold is one such triumph—an artificial intelligence program that has remarkable ability to predict protein 3D structures, showcasing the transformative potential of repurposing existing data (2). The prediction accuracy of AlphaFold relies heavily on the comprehensive raw and metadata from UniProt for protein sequences and the experimentally determined protein structures from the Protein Data Bank (3,4). Similarly, utilizing secondary genomics data was crucial for designing and optimizing the severe acute respiratory syndrome coronavirus 2 (SARS-CoV-2) vaccines (5). Again, metadata played a crucial role in tracking virus mutations, assessing vaccine efficacy and guiding public health strategies. In the realm of drug discovery, repurposing secondary omics data has been employed to uncover new uses for existing medications, offering valuable insights into disease mechanisms and drug interactions (6). These examples highlight the substantial impact of secondary omics data usage on research and underscore the importance of accurate and comprehensive metadata.

Transitioning into an era of data-driven knowledge extraction and *in silico* experimentation, the role of secondary data use will grow exponentially. The high-throughput nature of omics data renders it ideal for machine learning (ML) applications, facilitating robust model training and exploration of intricate patterns (7,8). There has been a recent influx of researchers using public data to build ML tools to address challenges in the field of omics research such as the prediction of peak intensities in tandem mass spectrometry experiments (9). The predictive power and interpretability of ML models rely heavily on the extent and quality of the data employed implicating the crucial importance of suitable primary data available for such use.

In this work, we evaluated the quality of omics data curation across public repositories to assess their suitability for secondary research. Collecting data at scale, from different repositories and sourced from different types of experiments, necessitated investigating high-level database features as well as dataset-specific characteristics. Within the focus of this study, these features were investigated for the test case of the human embryonic kidney cell line HEK293, and its derivative HEK293T as a model. Our compilation of a pan-omics dataset, exceeding 100 TB in size revealed several challenges, which are widespread and universally prevalent. These challenges were not database- or data domain-specific since the repositories under investigation accepted data regardless of species type or cell line. Our findings challenge the assumption that public repositories are reliable sources for secondary research, instead revealing them as potential hazards where data integrity is frequently compromised.

Reporting and highlighting these challenges, we underscore opportunities for data owners and stewards to enhance the reproducibility and longevity of experimental data, thereby facilitating value-added research through secondary use. Without urgent reform, omics data repositories risk becoming digital wastelands—rich in data but lacking actionable insights. All of this raises the critical question: what is the real value of omics data?

## Materials and methods

### Database selection

A comprehensive exploration of online omics databases was undertaken to retrieve data related to the human embryonic kidney cell lines HEK293 and HEK293T. The search keywords were ‘human embryonic kidney cell’ and ‘HEK’, and the relevant genomic, transcriptomic, epigenomic, proteomic and metabolomic datasets were identified from the following public databases: Sequence Read Archive (SRA) (10), European Nucleotide Archive (ENA) (11), PRIDE (12), jPOST (13), iProX (14), MassIVE (15), MetaboLights (16) and Metabolomics Workbench (17).

### Search protocols

The data search queries followed a logical order, initiating with the cell line of interest and adapting to the search flexibility of each database. The final search keywords are presented in Table 1. The data collection adhered to a methodical approach, spanning from December 2022 to May 2023.

**Table 1.** Breakdown of search keywords in each database according to omic layer

Omic layer	Repository	Search keywords					
Genomic	SRA	HEK293 OR HEK293T	*Homo sapiens*	Fastq	Illumina	Paired	DNA
	ENA		*Homo sapiens*	Genomic	Illumina		
Transcriptomic	SRA	HEK293 OR HEK293T	*Homo sapiens*	Fastq	Illumina	Paired	RNA
			*Homo sapiens*	RNA	Single cell		
	ENA		*Homo sapiens*	Transcriptomic	Illumina		
			*Homo sapiens*	Single cell	Illumina		
Epigenomic	ENA	HEK293 OR HEK293T	Methylation	Project			
			Phosphorylation	Project			
Proteomic	MassIVE	HEK293 OR HEK293T					
	iProX	HEK293 OR HEK293T	*Homo sapiens*				
	jPOST	HEK293 OR HEK293T					
	PRIDE	HEK293 OR HEK293T	*Homo sapiens*				
Metabolomic	MetaboLights	HEK293 cell					
	Metabolomics Workbench	HEK293					

### Metadata extraction

Metadata from SRA was directly extracted in bulk from the database with file type ‘run info’ and converted to .csv format. The same metadata features were manually selected, and bulk extracted from the ENA database in .tsv format. The extracted metadata was collated into a new database in .csv format. Details of the sequencing metadata collected are outlined in Supplementary Table S1. Metabolomics metadata was extracted manually from selected databases. Missing information in the metadata was gathered from the cognate publications and supplementary materials. Metabolomics metadata was compiled into .csv file format, with details available in Supplementary Table S1. Proteomics metadata was extracted in bulk from MassIVE in .tsv format. Additionally, manual extraction of proteomics data was conducted from PRIDE, iProX and jPOST databases. Any missing metadata was gathered from cognate publications. The extracted proteomics metadata was compiled into a .csv file, and the specifics are provided in Supplementary Table S1.

## Metadata evaluation

### Evaluation of metadata was conducted using the pandas version 2.0.3 and NumPy version 1.25.0 python packages for analysis

Missing values in the sequencing metadata were methodically identified and missing information was manually input into the metadata to ensure completeness. The missing information in metadata, which was bulk extracted from MassIVE, was filled in from cognate publications. For all metadata extracted, trends and abundances of missing metadata were evaluated, aiming to understand the patterns across the dataset.

Collected metadata was screened for inadequacies, including non-unique sample names and uninformative reporting in sequencing library strategies and selections, as well as uninformative methods of proteomic quantification.

Inaccuracies in collected metadata were assessed by investigating the reported gender, cell type and species associated with the data. Accurate species and cell sex identification in the metadata were selected as crucial parameters for dataset reliability. Incorrect assignments resulted in data removal, with inaccuracies in species or sex assignment flagged and eliminated. The manual nature of the metabolomics metadata collection process allowed the assessment of missing and inadequate metadata to proceed in tandem with data collection.

### Database establishment

The metadata was merged into a novel database comprising only the information of studies considered of acceptable and sufficient quality (data accession IDs provided in Supplementary Table S2). The abundances of each type of omics data were determined for HEK293 and HEK293T. The database was cross-checked for errors, especially those in which the dataset was misplaced in the wrong omic category based on the sequencing library strategy and library selection metadata available. Duplications within and across databases were removed. To address instances where databases allowed a single sample to be categorized across multiple omic layers, we used information based on the library strategy within the metadata. This enabled programmatic assignment to the correct sequencing data type, aligning with the sample preparation methodologies used. In cases where a study was associated with data involving more than a single cell type, manual curation ensued with a thorough review of associated publications. Subsequently, all studies were meticulously assigned to the correct cell line, eliminating any duplications.

### Raw data download

The SRA toolkit version 3.0.2 was used to automatically download the raw sequencing data. Raw metabolomics data were downloaded from the Metabolomics Workbench and MetaboLights using file transfer protocol (FTP). Raw proteomics studies were downloaded from PRIDE and MassIVE with FTP; to retrieve studies from iProX and jPOST, hypertext transfer protocol secure (HTTPS) was implemented.

### Repository feature evaluation

Each of the steps taken to compile the pan-omics dataset was evaluated and key repository features were assessed. The features include ‘search flexibility’, assessing the ease of refining database search functions for accurate study retrieval; high flexibility databases offer many options for search refinement including Boolean search, moderate search flexibility refers to databases that have options to refine the search but lack Boolean operators and those that have low flexibility provide minimal search parameter choices.

‘Metadata download flexibility’ evaluates the ease of extracting essential metadata, ranging from programmatically obtainable to manually obtainable options; high flexibility indicates easy extraction protocols that support bulk retrieval of study metadata, moderate flexibility suggests a less straightforward process with tools for metadata retrieval on a per study basis and low flexibility implies difficulty in obtaining the required metadata with no option for bulk retrieval. ‘Instances of missing metadata’ quantifies the frequency of missing metadata within the obtained dataset, relative to the amount of data for that database, the datatype and comparisons across the entire collected dataset; a high rating indicates a notable occurrence of missing metadata, a moderate rating suggests a moderate frequency and a low rating denotes infrequent or rare instances of missing metadata. ‘Instances of inadequate metadata’ pertains to metadata lacking sufficient descriptive information for making informed decisions about the collected data; a high rating suggests frequent instances where the available information is insufficient, a moderate rating indicates intermediate adequacy and a low rating implies that most of the obtained data are informative and sufficiently described. ‘Raw data download flexibility’ refers to the ease of retrieving raw omics data from each database; high flexibility rating suggests the availability of multiple download methods and/or simple and automated downloads, enabling the retrieval of data for multiple studies simultaneously, moderate rating implies the availability of a less straightforward downloading process, but with tools available for downloading multiple datasets that are associated with a single study at a time, or tools that are not optimized for bulk downloads and a low flexibility rating indicates a lack of advanced tools for raw data extraction, necessitating a manual, individual download for each study.

### System

The study utilized a Dell Precision 7920 Tower workstation with dual Intel^®^ Xeon^®^ Gold 5217 processors, 64 GB DDR4 ECC RAM and an NVIDIA^®^ RTX™ A4500 graphics card. Operating on Windows 10 Pro for workstations with Windows 11 Pro license, it featured PCIe NVMe SSDs and SATA HDDs for storage within the Precision 7920 Tower Chassis, facilitating comprehensive scientific analyses and data processing.

## Results and discussion

Constructing a structured dataset is crucial in utilizing pre-existing data effectively, as it substantially impacts the value of any secondary analysis. In this study, we compiled a pan-omics database for HEK293 and HEK293T from eight repositories (Table 2). These repositories varied in omics coverage as well as their capability to facilitate data retrieval for secondary use.

**Table 2.** Databases overview

Repository	Data type	Search flexibility	Metadata download flexibility	Instances of missing metadata	Instances of inadequate metadata	Raw data download flexibility
SRA	Sequencing^a^	High	High	Low	Moderate	High
ENA	Sequencing^a^	High	High	Low	Moderate	High
MetaboLights	Metabolomics	Moderate	Low	Low	Moderate	Moderate
Metabolomics Workbench	Metabolomics	Low	Low	Low	Low	Low
PRIDE	Proteomics	Moderate	Moderate	Low	Moderate	Moderate
iProX	Proteomics	Moderate	Low	Moderate	Moderate	Moderate
jPOST	Proteomics	Low	Low	Low	Low	Low
MassIVE	Proteomics	Low	High	Moderate	Moderate	Low

^a^Refers to genomics, epigenomics and transcriptomics data collectively.

**Figure 1.**
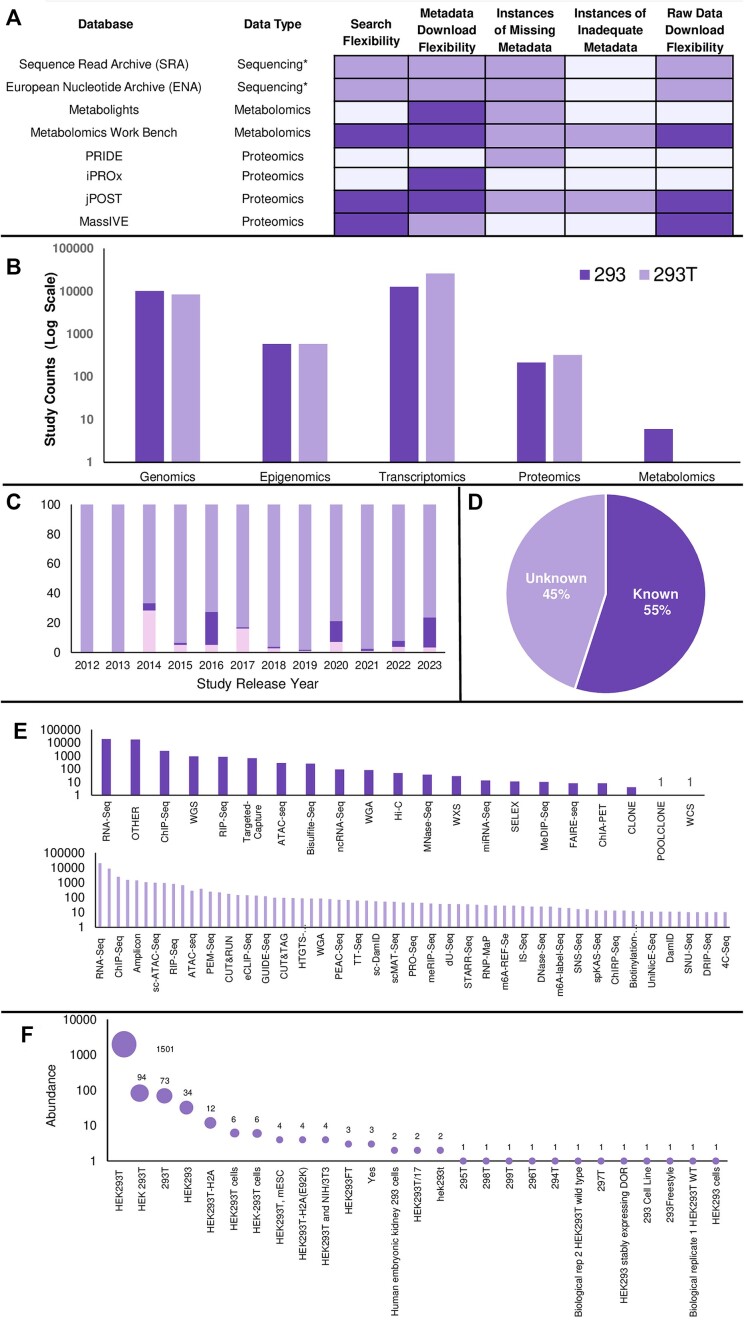
Assessment of the data curation process. (**A**) Overview of the issues observed across all databases. In the image, light purple indicates areas with high desirability, pale purple indicates intermediate desirability highlighting aspects needing improvement and dark purple signifies low desirability. The superscript * refers to genomics, epigenomics and transcriptomics collectively. (**B**) Relative abundances of different data types available for each cell type. Note the *y*-axis representation in log_10_ scale. (**C**) Reporting of sample sex on the SRA over time. The light purple colour indicates missing data. Notably, the inclusion of sample sex as an option began appearing around 2014, with the highest accuracy in identifying the HEK293 cell line as female, represented by the pink shade, on average. Dark purple shades represent instances labelled as ‘not applicable’ or ‘unknown’, offering valuable but imperfect insights. This dark purple shade indicates a recurring pattern where researchers frequently omit specifying sample sex when submitting data to the SRA. Since 2022, researchers have shown improvement in data descriptions, signalling a positive shift; though, accuracy enhancement remains crucial. (**D**) Insights into the quantification method of proteomics studies obtained from the PRIDE database. One hundred percent of studies sourced from PRIDE include a value for the method of quantification in the properties table on the study page. Almost half of these studies lack an informative method of quantification; 45% are marked as unknown highlighting a crucial gap in essential proteomics metadata. It is important to note that this missing information is available at the publication level for the majority of the datasets. (**E**) Choice of library strategies for sequencing data. Dark purple bar plot illustrates the distribution of library strategy types according to the metadata reported in the repositories, with the ambiguous reporting as ‘OTHER’ prompting the need for manual curation. A more comprehensive array of library strategies can be depicted following manual curation as shown in the light purple bar plot; library strategies with <10 occurrences were removed for clarity. (**F**) The frequencies and variances in the metadata description of the HEK293T cell line on the ENA. Each bubble represents a specific cell line description, with the size indicating the frequency of occurrence in descriptions.

We assessed eight repositories, including the SRA, ENA, MetaboLights, Metabolomics Workbench, PRIDE, iProX, jPOST and MassIVE, based on various end-user features and provided a comparative evaluation outlining this process (Table 2) and an assessment of the desirability of these capabilities (Figure 1A). It is important to emphasize that the ratings of ‘high’, ‘moderate’ and ‘low’ are subjectively assigned to compare the same feature across all databases. See the ‘Materials and methods’ section for detailed description of these evaluation features. These ratings served as relative measures enabling consistent comparative evaluation across all databases.

### Data discovery: search capabilities in scientific repositories

Data accessibility depends on the flexibility of the search capabilities of repositories. Sequencing repositories such as the SRA and ENA excel at offering advanced search options, which facilitate efficient extraction and retrieval of study data. MetaboLights, iProX and PRIDE provide reasonable search flexibility; however, databases such as Metabolomics Workbench, MassIVE and jPOST have limited query capabilities. Notably, jPOST lacks support for logical operators in search. An example to highlight the potential consequences of such lack of capability is evident for this cell line; all studies (100%) retrieved from the repository, when the search keyword HEK293 was used, were identical to those when the keyword HEK293T was used, thereby necessitating manual assignment of the retrieved data to the correct cell line, underscoring the pivotal role of search flexibility in ensuring relevance in data retrieval.

### Metadata access: navigating repository capabilities and constraints

After establishing specific search and retrieval protocols for each database, our focus turned to the investigation of metadata, in particular, to the downloadability of metadata for relevant studies across various omic layers. As with data retrieval, metadata extraction efficiency was shown to vary substantially across databases. Sequencing repositories enable rapid and easy download; SRA has fixed metadata options while ENA allows users to select metadata features for extraction, enhancing query customization. Advancements in sequencing technologies have made sequencing data more efficient and affordable to generate, directly influencing the wealth of information available in repositories that accept sequencing data. Additionally, the sequencing databases have been well established for several decades now, and consequently, they house considerably more data than their counterparts for proteomics or metabolomics data. This historically pioneering role has allowed them to develop superior submission protocols and adapt to the evolving needs of research communities more effectively than others (18,19). However, historical datasets often retain outdated metadata standards, which are not updated to reflect current best practices, and the exponential influx of dataset submissions annually overwhelms the capacity of data repository stewards to manage them effectively, hence lending the responsibility to the owners of the primary data.

Obtaining the required metadata from metabolomic databases was a labour-intensive process. The lack of any requisites for mandatorily submitting a structured metadata file for metabolomics data resulted in the submission of metadata into the repository in inconsistent formats, and from a secondary end-user perspective, this necessitated manual curation, occasionally involving resorting to associated publications, to gather all the necessary metadata.

Proteomics data extraction methods vary in accessibility and functionality across different databases. MassIVE provides a functional metadata extraction tool, allowing .tsv format export with column filtering. The PRIDE Archive has an application programming interface (API) that allows metadata extraction in JSON format but is limited by its lack of advanced filtering and verbose data output. Other features of the API provide metadata extraction; however, they require study accession codes that cannot be mass exported from PRIDE, limiting extraction accessibility. jPOST and iProX completely lack bulk metadata extraction capabilities, requiring manual curation by per-study retrieval. This varied accessibility to proteomics metadata is primarily caused by the fact that, despite nearly two decades since the establishment of proteomic databases, the field is still lacking an agreement on a standardized metadata format, which, in turn, hinders effective data management (20–22). 

There have been recent efforts to enhance research reproducibility by extending MAGE-TAB, originally designed for genomics, to capture proteomics sample metadata and experimental data. It aims to link data files to sample attributes, supporting the annotation of both technical and biological metadata, including factor values using ontology terms (23). A drawback of this format is the manual annotation with spreadsheet software. Manually populating spreadsheets often results in inadequate machine readability and inconsistencies, as demonstrated by the consistent gene name errors (24,25). Perez-Riverol (23) actively encourages community input on the proposed sample and data relationship format (SDRF) for proteomics metadata format, fostering collaborative strides in addressing these persistent challenges. To support this initiative, members of the European Bioinformatics Community for Mass Spectrometry launched a community wide initiative through GitHub to annotate public datasets according to the SDRF format. They have annotated >200 proteomic datasets. Despite this effort, only a small percentage of datasets include submitter provided SDRF annotations and the overall percentage of SDRF annotated projects remains low at 3.9% by mid-2022, as reported by Claeys *et al.* (26).

It is of note that PRIDE stands out as the only one of the four proteomics repositories in our study that is requesting an SDRF file at data submission (27). To effectively reuse a PRIDE dataset, researchers need to navigate the repository data and review associated publications, including their supplementary information, to obtain a complete metadata picture, all while being vigilant for contradictions between available sources. To mitigate these challenges, lesSDRF, a user-friendly tool aimed at streamlining the annotation of proteomic datasets, was developed to encourage primary data users to submit their metadata before or at the time of raw data submission (26). These efforts are most welcomed by the secondary data user community; however, they are not scalable and the lack of robust metadata in the field is hindering progress with proteomics research.

Skewness in data availability, in other words, the dominance of a particular type of data in a heterogeneous database, needs careful consideration prior to any secondary analysis. Imbalanced or disproportionate availability of different types of data may present challenges for secondary data analysis, necessitating employment of dedicated data handling approaches. Emerging technologies could create obstacles in data analysis due to the unique types of data they generate. Additionally, when exploring novel organisms or new applications for existing ones, researchers may inadvertently prioritize certain types of data over others, potentially leading to biases in data collection and analysis as demonstrated here by the noticeable skewness in the number of datasets available for each data type for HEK293 cells (Figure 1B). The compiled HEK293 omic database was dominated by sequencing datasets (99%) with a breakdown of genomic (∼31%), epigenomic (∼2%) or transcriptomic (∼66%) datasets. Proteomic dataset availability was modest in the dedicated repositories (<1%) and available metabolomic datasets were very limited (∼0.01%).

While incorporating diverse biological information typically enhances the informative capabilities of secondary analysis, it is crucial to utilize existing data resources appropriately to avoid bias and misinterpretation of results. An understanding of where the abundance bottlenecks lie, through secondary or meta-analysis of the current data landscape, may also provide primary users and data owners clear insight as to the future opportunities in generating novel insight. It should also be noted that since the available metabolomics data were negligible for HEK293 cells in comparison with other data types; the challenges around the collection of metabolomics data discussed previously should be carefully assessed with respect to their generalizability.

### Mind the gap: impact of missing metadata on research efficiency

Another challenge associated with the reuse of data was the missing or incomplete information in metadata accompanying the datasets in the repositories. This problem manifests itself as a dual challenge of absent or inadequately reported metadata. Gould *et al.* (28) re-emphasize the importance of standardized metadata as a solution to overcome this challenge. In this reported study, over 200 ecologists arrived at different conclusions when analysing the same dataset, underscoring the impact that choices made during data analysis can have on the results even when provided with identical metadata. The consistent availability of metadata remains a critical concern in scientific research, potentially offering compounded challenges around reproducibility of analysis results. The absence of such information begs the question: is it due to the absence of standards or the lack of adherence to existing ones by primary users or repositories?

The issue of varied metadata standards across omics data types is well documented, as highlighted by a 2013 paper addressing the challenges around developing standards for such complex evolving data across an array of research domains (29). The challenge of metadata standards extends beyond single species omics to the realm of metagenomics in microbiome research. Recognizing this challenge for meta-omics, Cernava *et al.* (30) proposed for the development of a globally recognized metadata submission tool adhering to the FAIR (findable, accessible, interoperable and reusable) guiding principles. Researchers’ adherence to defined standards warrants further scrutiny. In 2020, the Genomic Standards Consortium stressed the critical need for FAIRness in contextual genomics data. This arose from challenges leveraging existing genomics data during the pandemic. The article addressed metadata issues related to Coronavirus disease (COVID-19), specifically how it impacted research efficiency, and emphasized the importance for researchers to invest time in accurately describing their data. The work reinforced the importance of selection of suitable metadata, integration of standardization into workflows, prioritization of community-driven initiatives and crafting of user guidelines (31). While standardization does not aim to impose uniformity in the generation or interpretation of the data, it was proposed to reduce variation and enhance the reliability and reproducibility of the scientific process, maintaining consistency (32). As we observe in these efforts, overcoming metadata challenges requires collective effort and responsibility of the scientific community.

Within data repositories, ENA offered enhanced metadata extraction flexibility when compared with SRA; however, it has a higher frequency of missing metadata, partly due to varied submission requirements. Metadata from ENA lacks sequencing load dates (100%), file sizes (3%), library names (59%), sample names (5%), sample sex (85%) and centre names (47%) across the extracted datasets. SRA exhibits fewer instances of missing metadata, primarily lacking non-mandatory library names (78%) and often featuring missing or uncertain information on sample sex (91%) (Figure 1C). Bhandary *et al.* (33) conducted a study investigating over 2300 paired-end Illumina coding RNA-seq runs from *Zea mays* and reported that ‘troubling patterns’ in metadata quality were observed, mirroring the issues that we observed in the data pertaining to HEK293 and HEK293T cells. In the SRA, they found that 27% of the 61 examined metadata fields consistently lacked entries, with an additional 37% just partially completed. Only 29% of fields were consistently filled in by data submitters, with 10% being auto generated.

Despite manual collection, metabolomics studies exhibit pervasive missing metadata, both within the database and in the cognate publications. Notably, the mass-to-charge ratio (*m*/*z*) is absent in 60% of the acquired metabolomics studies utilizing mass spectrometric approaches, hampering both data reanalysis and experimental repeatability of the results in another laboratory. To gather all essential metadata for these studies, it is often required to convert the raw metabolomics spectral files to mass spectrometry markup language and extract the metadata, which is not an efficient and, in most instances, an unfeasible process.

Aggregating metadata from five proteomics data sources naturally led to substantial information gaps, including missing species and specific makes and models of mass spectrometry instruments. More importantly, a critical metadata feature absent in several proteomics studies is the method of quantification. Over 45% of studies collected from the PRIDE database list the quantification method as unknown, presenting a significant challenge to study reproducibility and result validation (Figure 1D). LesSDRF developers, too, found widespread metadata deficiencies in >200 PRIDE projects and the related research articles. They, too, found that this issue extends across all proteomics repositories in the ProteomeXchange, a data-sharing initiative in the field of proteomics (34). Incomplete protein labelling methods and protein modifications and shared discrepancies between article and PRIDE metadata were prevalent, particularly concerning tissue annotation. PRIDE is often missing detailed annotations, relying on broad terms such as ‘cell culture’ (26). Despite these metadata inadequacies, PRIDE stands out as a major contributor to the ProteomeXchange hosting over 28 000 datasets, in comparison iProX has nearly 4000 and MassIVE and jPOST each contribute >2000 datasets as of July 2024.

### The bare necessities: how errors and ambiguity undermine research quality

In addition to insufficient metadata, inaccuracies were identified including cases where samples were erroneously labelled. One such example was tagging of the widely known female HEK293 cell line as male. To explore this further, we analysed the trends in sex metadata within the SRA database over the years (Figure 1C). It was observed that sex was first included as a reportable metadata feature in 2014, with this year being the same year that the most researchers correctly described the gender of the cell line in their submissions. Over the years, we note that researchers increasingly omitted the reporting of sample sex for the majority of data reported on HEK293 cells. While this does not have a major impact on secondary use of these data specifically, it does call into question the credibility of the other reported metadata of the associated study. In other areas of research where gender has a greater impact, such as health omics with patient samples, this issue of haphazard sample sex reporting would be concerning, such as in the investigation of the role of sex, or as previously reported in instances where omics analysis revealed sex-based differences in disease characterization (35,36). Bond *et al.* (37) called for a mandate of data sex annotation to enable sex specific analysis and to progress towards accounting for the biology of sex differences. In the evaluation of HEK293 data, we see an encouraging trend, since 2021 of increased sample sex annotation in SRA; however, there is still ample opportunities for data submitters to enhance precision.

Additionally, we observed a striking occurrence of non-unique sample names (43%) during the investigation of the sequencing data. This problem was not exclusive to differences between studies, where >1% of sample names overlapped, for example the use of sample names such as ‘HEK293’ or ‘HEK293_WT’ occurred across studies. It was predominately an issue within individual studies, with 99% of sample names being non-unique, such as multiple samples being named with the same GEO accession study number. Consequently, the presence of non-unique sample names significantly affected the clarity and informativeness of the names within datasets.

The library strategy types were also among information reported ambiguously as a number of submissions tagged the library strategy as ‘other’ and avoided the provision of further details (Figure 1E). Just >33% of sequencing runs categorized sequencing library strategies as ‘other’, 35% categorized sequencing selections as ‘other’ and 32% of the runs with both library selection and strategy as ‘other’. This oversimplified categorization lacks crucial details about how the sequencing libraries were prepared, impeding informed decision-making and compromising the reproducibility of analyses and experimentation in various research endeavours. Moreover, the association with different sequencing libraries implies that distinct sequencing studies may pertain to diverse omic layers, significantly impacting how the bioinformatics-driven re-analyses would be carried out. For our purposes, we obtained the correct library strategy from the associated abstract available within the repositories or from the cognate publications. There were still instances (<10%) where the library strategy was unclear from the associated publication impeding study replication.

In genomic studies of HEK293T cells, diverse and inconsistent descriptions of the cell line name, including numeric and capitalization variations, were observed and complicated metadata evaluation (Figure 1F*)*. A simple solution to address this challenge would be the introduction of a drop-down selection menu with predefined, consistent cell line names for enhanced clarity and streamlined metadata assessment in the submission portals of public repositories. While some errors could be prevented through simplified metadata upload procedures or increased awareness of data generation best practices, random errors will persist. The complete elimination of errors is unlikely, as evidenced by the ongoing challenges and retractions in scientific research, with 2023 as the year with a record number of research article retractions (38).

Bernstein *et al.* (39) introduced MetaSRA, a database designed to improve the poorly structured metadata within the SRA. MetaSRA normalizes this metadata using an ENCODE-inspired schema, mapping samples to biomedical ontologies, categorizing sample types and extracting properties. This automation facilitates large-scale analysis of biomolecular processes and phenotypes across diverse diseases, tissues and cell types, enhancing querying for relevant samples. While currently focusing on human RNA-seq samples from the Illumina platform, MetaSRA aims to include samples from other species and assay types in future iterations, which could prove beneficial across different organisms and experimental systems.

In methods utilized for secondary analysis of data such as ML, consistent and accurate data are crucial for machine understanding, yet inconsistent naming conventions in databases such as ENA and SRA were reported to pose challenges (40). There are identical metadata entries, such as the ‘BioProject accession code’ in SRA matching ‘study accession’ and ‘study alias’ in ENA. Reconciling inconsistencies in describing identical metadata features across databases requires meticulous quality control. Consistent naming conventions and the use of ontologies would improve data organization and enhance interoperability (33). Managing the expanding volume of biological data requires addressing overlapping ontologies. Initiatives such as the Open Biological and Biomedical Ontologies project aim to organize ontology development based on shared standards, enhancing accessibility and reuse (41). Nevertheless, they have not yet been taken up efficiently in enabling (meta)data sharing across platforms and repositories.

Achieving high precision and recall in database searches relies on the uniqueness of datasets. Comprehensive metadata ensures that uniqueness can be tested, and is necessary to enhance findability, ensuring relevant datasets are accurately identified and retrieved. In this particular project, there were instances of duplicated sequencing runs, which were collected for more than one omic layer for both cell lines, meaning that based on the associated metadata, certain datasets were pulled as transcriptomic *and* epigenomic *and* genomic. Specifically, in data concerning the HEK293 cells, 20% of the runs were duplicated across the transcriptomics and epigenomics data collected and ∼30% of the runs were duplicated across the transcriptomics and epigenomics data for HEK293T cells. These duplications necessitate reviewing of additional metadata to ensure that the data are appropriately assigned to the correct omics data type for subsequent analysis. Due to the array of metadata errors as well as the duplications across HEK293 and HEK293T cell lines observed in proteomics studies, it was prudent to investigate whether there were duplications across sequencing datasets compiled for both cell lines. Over 2% of runs were duplicated for both HEK293 and HEK293T cells, necessitating reassessment for accurate cell line assignment.

### Efficient data access: evaluating the ease of raw data acquisition across platforms

Among the platforms explored, the SRA toolkit proved efficient for acquiring raw sequencing data, offering user-friendly access, dataset retrieval, file format conversion and quality control. While both SRA and ENA provide numerous tools for obtaining raw data, the SRA toolkit stands out for its efficiency and ability to interact with both repositories. Additionally, it supports background and cloud-based operations. In contrast, proteomic databases primarily rely on methods designed for single-study retrieval, such as and FTP and HTTPS, causing bulk raw data extraction to be impractical. While both PRIDE and iProX support bulk downloads via the Aspera client, the need for a manually compiled dataset list limits its effectiveness, rendering it ineffective for bulk retrieval. Python packages such as pridepy, designed to facilitate bulk downloads, face performance challenges and lack active maintenance, hindering their effectiveness for intended use.

### From generation to reuse: enhancing omics data quality

Our findings underscore the need for enhanced data management practices to ensure that public repositories can support impactful secondary research. However, despite the challenges observed, there are cases where data from these repositories have been effectively reused to develop advanced research tools. Tools such as Kraken have been developed, using data from SRA, and have had significant impact on microbiome studies and the identification of pathogens (42,43). Similarly PeakBot‘s development to better detect chromatographic peaks, utilizing ML and existing metabolomic datasets, from both MetaboLights and the Metabolomics Workbench, showcases how secondary data analysis can refine research tools (44). Datasets from the ProteomeXchange have been employed by quantms, an open-source cloud-based pipeline that outperforms MaxQuant, a gold standard in proteomics research, in processing speed and protein quantification, enabling a more efficient and reproducible proteomics analysis (45). By improving data analysis capabilities and streamlining workflows, these tools exemplify how leveraging secondary data is crucial for driving innovation in the omics ecosystem. However, these achievements rely on high-quality, well-curated data.

The real value of omics data is vast and potentially limitless. By embracing a circular economy approach, where data are treated as a reusable resource rather than a one-time asset, we can create a sustainable model that continually fuels new discoveries and innovations. Central to this economy is metadata, crucial for making omics data accessible, understandable and reusable across research domains. Standardized metadata enhances the integration of data from diverse sources, leading to novel cross-disciplinary insights and improved research tools and algorithms. This facilitates the reuse and repurposing of data, thereby maximizing its value beyond its initial application. In addition, well-managed metadata helps identify and address bottlenecks in data generation processes. By highlighting recurring issues, metadata can inform the refinement of data-generation technologies and enhance overall research efficiency. With comprehensive and standardized metadata, omics data would not only maintain its extensive value but also evolve continuously, offering limitless opportunities for scientific progress and technological innovation.

In this era of big data and ML, acquiring extensive data is crucial, prompting this investigation of omics data accessibility through the search and download processes together with the completeness of the associated metadata. While we focused on HEK293 and its derivative HEK293T, specific cell lines essential to advanced therapies, the challenges encountered in data collection for these cell lines extend beyond this specific field and species. Navigating these and other similar complexities is vital for researchers to advance their investigations and improve data management and accessibility in the broader scientific community. Aligned with our commitment to open data and science initiatives, researchers should continuously improve practices to support public databases in line with their intended use. Achieving this heavily relies on widespread adoption of robust and standardized protocols for data and metadata sharing by primary data users, fostering effective knowledge sharing and accelerating discoveries in diverse research domains.

There is no universal solution to the highlighted challenges of the secondary use of publicly available omics data, as both the data and databases vary widely. The inherently interdisciplinary nature of omics research means that researchers from different domains, such as computational biology, experimental science and clinical research, each have distinct requirements for data management. For example, a computational biologist may prioritize quick access to high volume data retrieval for ML, while an experimental biologist might prioritize usability and reproducibility. A solution for one group of researchers may not be necessarily applicable to another. Ultimately, initiation of a dialogue that leads to collaborative improvements in data management systems, ensuring that all users—regardless of their expertise—benefit from more accessible and reliable data is imperative. Ideally, this will also encourage primary data submitters to take greater care in their work, while highlighting areas that database stewards may find actionable insights. Nevertheless, there are several improvements that could benefit both advanced users focused on large-scale data mining and non-experts seeking clear and easy to use datasets. One key area for improvement is metadata.

Metadata is often underestimated, what one person sees as metadata another might view as data, implicating huge potential. Effective data practices are essential for producing high-quality research and maintaining scientific integrity. For this reason, metadata is fundamental not supplementary. It supports accurate documentation, reproducibility and accountability, ensuring that research findings can be verified and built upon. Mastering data generation, management and evaluation—including addressing issues such as missing or inadequate metadata—is crucial for enhancing the credibility and impact of research. By adopting robust data practices and prioritizing thorough metadata management, researchers improve the reliability of their findings, contribute to the longevity and utility of their data within the scientific community, and elevate their own professional reputation (46).

The real value of well-curated omics data was demonstrated in 2020 by a collaborative effort involving 25 researchers from four countries in a ‘datathon’ aimed at recovering and curating missing metadata associated with sequencing studies. More than 2000 hours were dedicated to this task, culminating in the restoration of data for secondary reuse estimated to be worth over £1.6 million (47). This undertaking underscores the immense financial investment required to make omics data usable and valuable for future research. More importantly, it highlights the critical role of effective data management from initial submission phase to enable resource effective secondary utilization. The true value of omics data can only be realized when metadata is robust.

The exponential growth in data made publicly accessible presents opportunities and challenges for scientific research and high-throughput omics data are no exception. However, this opportunity materializes with the responsibility to understand and comply with data integrity and adopting sensible maintenance practices. Ensuring the accuracy and completeness of historical data presents a formidable task within our community. While there have been numerous tools and initiatives developed to mitigate the deficiencies in metadata quality, the primary responsibility for maintaining high data quality ultimately rests with the original data submitter. Tools alone cannot rectify inaccurate annotation without submitter engagement. To reinforce this responsibility, databases can adopt proactive measures such as highlighting instances of missing metadata in submissions and sending reminders to rectify them. Implementing a visual warning system on data access pages, indicating metadata availability status with colours such as green for high availability, amber for moderate and red for poor, can further incentivize submitters to prioritize metadata completeness. Moreover, implementing a peer review system for secondary users to flag data issues could serve as an additional layer of quality control. In cases where flagged issues remain unaddressed over time, they could be escalated to draw the attention of data stewards. To ensure high-quality metadata in the future, it is imperative for journals, data repositories and primary data users to collaboratively implement responsible practices, encourage community engagement and prioritize data integrity.

## Conclusion

Public repositories, primarily funded by taxpayers, serve as invaluable resources for the scientific community, providing access to vast amounts of data crucial for research and innovation. However, the increasing pace of technological advancement coupled with the reduction in data generation costs has led to a significant influx of data being submitted to these repositories. This leaves the available platforms with reduced capacity for rigorous checkpoints to be placed to scrutinize the completeness of metadata for each dataset submitted. This would in no way present itself as a realistic expectation in the current landscape of overwhelmed and underfunded repositories. As primary data generators, it is incumbent upon us to recognize our responsibility in ensuring the quality and integrity of the data we submit to these repositories. Just as research outputs are meticulously prepared and published in academic journals, similar strict standards must be adhered to when sharing raw data. Doing so not only upholds the integrity of our research but also has a profound impact on our legacy as members of a research community. The raw data we generate holds immense potential, serving as a foundation for future discoveries and innovations. By publishing these data to the highest standards, we not only contribute to the advancement of science but also foster a culture of transparency, collaboration and progress in scientific research. If we fail to improve our practice, it raises a crucial question: what is the real value of omics data?

## Supplementary Material

gkae901_Supplemental_File

## Data Availability

The datasets analysed during this study are available in the European Nucleotide Archive (https://www.ebi.ac.uk/ena/browser/home), Sequence Read Archive (https://www.ncbi.nlm.nih.gov/sra), iProX (https://www.iprox.cn/), jPOST (https://globe.jpostdb.org/), MassIVE (https://massive.ucsd.edu/ProteoSAFe/static/massive.jsp), MetaboLights (https://www.ebi.ac.uk/metabolights/), Metabolomics Workbench (https://www.metabolomicsworkbench.org/) and PRIDE (https://www.ebi.ac.uk/pride/). The list of project IDs is provided as Supplementary Table S2.
