# Supplementary material for: What is the real value of omics data? Enhancing research outcomes and securing long-term data excellence
Source: Nucleic Acids Res. 2024 Oct 17;52(20):12130–40. doi: 10.1093/nar/gkae901 (PMC11551742; doi:10.1093/nar/gkae901)
Supplement: gkae901_Supplemental_File [file gkae901_supplemental_file.pdf]

Supplementary Material

What is the *real* value of omics data? Enhancing research outcomes and securing long-term data excellence

Eva Price[1], Felix Feyertag[2], Thomas Evans[2], James Miskin[2], Kyriacos Mitrophanous[2], Duygu Dikicioglu[1*]

[1] Department of Biochemical Engineering, University College London, Gower Street, London, WC1E 6BT, UK

[2] Oxford Biomedica (UK) Ltd., Windrush Court, Transport Way, Oxford, OX4 6LT, UK

*Corresponding Author: d.dikicioglu@ucl.ac.uk

Contents

Supplementary Table 1 Metadata Obtained for Sequencing, Metabolomics, and Proteomics Data 1

Supplementary Table 2 Study Data Accession Codes 3

Supplementary Table 1 Metadata Obtained for Sequencing, Metabolomics, and Proteomics Data

Metadata Description	Explanation
Sequencing Data	
Bases	Total number of bases in sequence
BioProject	BioProject ID
BioSample	Single BioSample ID
Cell Line	Cell line for study
Center Name	Lab where data was generated
Database	Data obtained from
Download path	Link for direct download of sequence
Experiment	Experiment number
Lab Head	Principal Investigator
LibraryLayout	Describes the arrangement of fragments within a sequencing library
LibrarySelection	Method of obtaining specific fragments before sequencing
LibraryStrategy	Plan and approach for preparing the sequencing library
LibrarySource	Indicates the biological origin of the RNA or DNA used for sequencing
Model	Model of sequencing instrument
Platform	Brand of sequencing instrument
ProjectID	Unique project number

Publication	Study DOI
ReleaseDate	Release date for data
Run	Sequence run number
Sample	Sample accession number
SampleName	Sample name
Sex	Sex of sample
Spots	Number of reads
SRAstudy	SRA study accession number
Size_mb	Size of run in megabytes
Metabolomics Data	
Accession number	Study accession number
Control Used	Control conditions used in study
Database	Data obtained from
Publication	Study DOI
File Type	File types available for study
Ionization type	Ionisation method for the study
Lab Head	Principal Investigator
Mass Range	The mass range of the metabolites
Raw Data	Available only if there is raw data available for the study
Resolution	Assessed by FWHM (Full Width at Half Maximum), gauges the instrument's ability to distinguish features
Size GB	Size of study in gigabytes
Study Title	Name of the study
Targeted/ Untargeted	Shows whether the study is targeted or untargeted
Technique used for data generation	Instrumental technique used to generate metabolomic data
Proteomics Data	
Accession Code	Unique study accession code
Acquisition Method	Method for selecting precursor ions and fragmentation in mass spectrometry analysis
Analysis Type	Approach to analyse sets of analytes or molecular features within a sample
Announced date	Data of publication
Complete or Partial	Study completeness
Data set Identifier	Unique dataset identifier in ProteomeXchange
Database	Data obtained from
File Type	File types available
Instrument	Instrument model used to generate proteomic data
Key Word	Key words linked to study
Lab Head	Principal Investigator
Publication	Study DOI
Quantification	Method of quantification
Size GB	Size of study in gigabytes

Species	Species for study
Study Title	Name of the study

Supplementary Table 2 Study Data Accession Codes

Data Repository	Data Accession		
Sequence Read Archive	PRJNA834973	PRJNA816418	PRJNA798537
	PRJNA972627	PRJNA743687	PRJNA817000
	PRJNA972367	PRJNA893547	PRJNA811252
	PRJNA979982	PRJNA893548	PRJNA761932
	PRJNA972524	PRJNA890016	PRJEB47501
	PRJNA996618	PRJNA895169	PRJNA756903
	PRJNA972551	PRJNA853525	PRJNA809126
	PRJNA957096	PRJEB55318	PRJNA817018
	PRJNA915940	PRJEB54733	PRJNA789761
	PRJNA1004865	PRJNA882573	PRJNA779199
	PRJNA971099	PRJNA817825	PRJNA772898
	PRJNA975160	PRJNA862731	PRJNA700693
	PRJNA984170	PRJNA868858	PRJNA773803
	PRJNA984169	PRJNA798016	PRJNA707486
	PRJNA986884	PRJNA872331	PRJNA770365
	PRJNA911799	PRJNA862539	PRJNA720833
	PRJNA755299	PRJNA863493	PRJNA771464
	PRJNA973234	PRJNA635361	PRJNA773240
	PRJNA975432	PRJNA807606	PRJNA769512
	PRJNA992096	PRJNA870593	PRJNA769747
	PRJNA980726	PRJNA720613	PRJNA769533
	PRJNA923001	PRJNA802977	PRJNA768631
	PRJNA974390	PRJNA871245	PRJNA765915
	PRJNA972834	PRJNA871961	PRJNA728500
	PRJNA972535	PRJNA872329	PRJNA762516
	PRJNA972537	PRJNA825584	PRJNA735408
	PRJNA972528	PRJNA872334	PRJNA762984
	PRJNA985249	PRJNA861237	PRJNA761773
	PRJNA972497	PRJNA872347	PRJNA763568
	PRJNA972495	PRJNA872353	PRJNA763870
	PRJNA972550	PRJNA872367	PRJNA762160
	PRJNA973981	PRJNA799250	PRJNA768303
	PRJNA970104	PRJNA721271	PRJNA758206
	PRJNA970076	PRJNA858683	PRJNA661167
	PRJNA972343	PRJNA849262	PRJNA731307
	PRJNA973011	PRJNA859372	PRJNA737283
	PRJNA942467	PRJNA821514	PRJNA735610
	PRJNA943545	PRJNA822797	PRJNA723137
	PRJNA874457	PRJNA826521	PRJNA728251
	PRJNA894938	PRJNA827770	PRJNA680046
	PRJNA946328	PRJNA828159	PRJNA741106

	PRJNA869750	PRJNA705187	PRJNA725249
	PRJNA911336	PRJNA838014	PRJNA63443
	PRJNA937687	PRJNA758047	PRJNA746292
	PRJNA898625	PRJNA842559	PRJNA748082
	PRJNA915236	PRJNA793268	PRJNA721457
	PRJNA929529	PRJNA734898	PRJNA723634
	PRJNA774044	PRJNA847165	PRJNA723636
	PRJNA842564	PRJNA738959	PRJNA723824
	PRJNA854190	PRJNA853263	PRJNA726718
	PRJNA911577	PRJNA853268	PRJNA727322
	PRJNA911798	PRJNA858014	PRJNA731475
	PRJNA914300	PRJNA858013	PRJNA733174
	PRJNA915471	PRJNA858779	PRJNA734350
	PRJNA916665	PRJNA810334	PRJNA668782
	PRJNA780303	PRJNA850798	PRJNA739668
	PRJNA925176	PRJNA819982	PRJNA739670
	PRJNA937215	PRJNA860913	PRJNA739982
	PRJNA860573	PRJNA854675	PRJNA664751
	PRJNA945099	PRJNA853293	PRJNA756912
	PRJNA871035	PRJNA812697	PRJNA752948
	PRJNA951689	PRJNA835701	PRJNA731611
	PRJNA962057	PRJNA822038	PRJNA725675
	PRJNA943422	PRJNA805019	PRJNA747989
	PRJNA961317	PRJNA847381	PRJNA713914
	PRJNA930411	PRJNA850677	PRJNA704478
	PRJNA954164	PRJNA770428	PRJNA758251
	PRJNA895582	PRJNA793002	PRJNA692623
	PRJNA937454	PRJNA783665	PRJNA729585
	PRJNA907921	PRJNA802707	PRJNA720932
	PRJNA949853	PRJNA795197	PRJNA702984
	PRJNA917081	PRJEB47433	PRJNA702488
	PRJEB57975	PRJNA753136	PRJNA689546
	PRJNA759137	PRJNA818698	PRJNA680170
	PRJNA907774	PRJNA768458	PRJNA691552
	PRJNA888859	PRJNA213579	PRJNA587328
	PRJNA906920	PRJNA628862	PRJNA646210
	PRJNA967838	PRJNA786208	PRJNA612584
	PRJNA967688	PRJNA787568	PRJNA692762
	PRJNA821929	PRJNA787927	PRJNA679040
	PRJNA871232	PRJNA791602	PRJNA680290
	PRJNA835113	PRJNA796352	PRJNA657637
	PRJNA905048	PRJNA728901	PRJNA631681
	PRJNA900081	PRJNA798755	PRJNA635405
	PRJNA901977	PRJNA803153	PRJNA686651
	PRJNA898646	PRJNA805505	PRJNA686652
	PRJNA848090	PRJNA809653	PRJNA686653
	PRJNA896967	PRJNA771384	PRJNA687761

	PRJNA799878	PRJNA817206	PRJNA688075
	PRJNA882795	PRJNA782085	PRJNA689216
	PRJNA881616	PRJNA732565	PRJNA690776
	PRJNA893560	PRJNA811956	PRJNA691936
	PRJNA885970	PRJNA756785	PRJNA645936
	PRJNA879077	PRJNA796642	PRJNA705864
	PRJNA879340	PRJNA745452	PRJNA713336
	PRJNA883650	PRJNA787708	PRJNA719212
	PRJNA887059	PRJNA780310	PRJNA676022
	PRJNA887057	PRJNA805249	PRJNA719099
	PRJNA888075	PRJNA801688	PRJNA683925
	PRJNA888074	PRJNA785066	PRJEB39004
	PRJNA675830	PRJNA504541	PRJNA623395
	PRJNA667521	PRJNA506020	PRJNA636394
	PRJNA630828	PRJNA513013	PRJNA637776
	PRJNA637588	PRJNA511715	PRJNA639520
	PRJNA633116	PRJNA445210	PRJNA649280
	PRJNA666293	PRJNA433660	PRJNA671710
	PRJNA624815	PRJNA433929	PRJNA671709
	PRJNA603010	PRJNA475277	PRJNA681143
	PRJNA647636	PRJNA436448	PRJNA682225
	PRJNA649296	PRJNA443149	PRJNA270853
	PRJNA486548	PRJNA454744	PRJNA684509
	PRJNA598118	PRJNA477645	PRJNA686419
	PRJNA637247	PRJNA508094	PRJNA695451
	PRJNA638561	PRJNA474739	PRJNA721418
	PRJNA640821	PRJNA507860	PRJNA602701
	PRJNA642789	PRJEB26955	PRJNA733150
	PRJNA643545	PRJEB27856	PRJNA736239
	PRJNA648873	PRJEB28742	PRJNA737125
	PRJNA656844	PRJNA454765	PRJNA737127
	PRJNA658494	PRJNA454824	PRJNA740029
	PRJNA674902	PRJNA503988	PRJNA742550
	PRJNA604872	PRJNA482053	PRJNA491943
	PRJNA670368	PRJNA359383	PRJNA779491
	PRJNA674944	PRJNA413624	PRJNA801706
	PRJNA658855	PRJNA314722	PRJNA808389
	PRJNA656176	PRJNA386320	PRJNA810044
	PRJNA629763	PRJNA354073	PRJNA665240
	PRJNA629762	PRJNA387654	PRJNA826894
	PRJNA666282	PRJNA397048	PRJNA830897
	PRJNA666273	PRJNA368958	PRJNA841973
	PRJNA655949	PRJNA322340	PRJNA848234
	PRJNA622564	PRJNA347518	PRJNA849940
	PRJNA623461	PRJNA349456	PRJNA854664
	PRJNA476544	PRJNA357195	PRJNA884408
	PRJNA616316	PRJNA338747	PRJNA884407

	PRJNA622484	PRJNA377168	PRJNA889775
	PRJNA625049	PRJNA379976	PRJNA896596
	PRJNA606250	PRJNA380920	PRJNA925495
	PRJNA627176	PRJNA387005	PRJNA951253
	PRJNA660556	PRJNA398222	PRJNA951252
	PRJNA616114	PRJNA399514	PRJNA958234
	PRJNA622835	PRJNA399759	PRJNA961094
	PRJNA591007	PRJNA403847	PRJNA975834
	PRJNA623032	PRJNA419954	PRJNA978064
	PRJNA575340	PRJNA420568	PRJNA985777
	PRJNA623926	PRJNA381704	PRJNA991955
	PRJNA553240	PRJNA330894	PRJNA684754
	PRJNA609122	PRJNA422341	PRJNA701496
	PRJNA596996	PRJNA419407	PRJNA633957
	PRJNA598461	PRJNA351248	PRJNA736246
	PRJNA603357	PRJNA316460	PRJNA635270
	PRJNA589228	PRJNA317714	PRJNA675418
	PRJNA604267	PRJNA419990	PRJNA876837
	PRJNA604277	PRJNA361425	PRJNA642884
	PRJNA604281	PRJNA399227	PRJNA630002
	PRJNA601178	PRJNA399616	PRJNA590635
	PRJNA602958	PRJEB20596	PRJNA592253
	PRJNA603565	PRJNA318578	PRJNA593843
	PRJNA604410	PRJNA361443	PRJNA750488
	PRJNA607539	PRJNA254528	PRJNA817034
	PRJNA612330	PRJNA183346	PRJNA731544
	PRJNA604677	PRJNA183941	PRJNA610420
	PRJNA604851	PRJNA188409	PRJNA762559
	PRJNA608802	PRJNA213263	PRJNA628100
	PRJNA544395	PRJNA217298	PRJNA743070
	PRJEB35808	PRJNA217765	PRJNA801869
	PRJEB36466	PRJNA224140	PRJNA727693
	PRJNA606866	PRJNA233403	PRJNA600132
	PRJNA531587	PRJNA252938	PRJNA675913
	PRJNA609749	PRJNA265199	PRJNA678030
	PRJNA595860	PRJNA278742	PRJNA675696
	PRJNA596353	PRJNA279892	PRJNA732942
	PRJNA561932	PRJNA279961	PRJEB51351
	PRJNA561027	PRJNA284156	PRJEB36717
	PRJNA551796	PRJNA284251	PRJEB36735
	PRJNA548306	PRJNA301900	PRJEB37897
	PRJNA575526	PRJNA301999	PRJEB34618
	PRJNA528767	PRJNA309545	PRJNA613894
	PRJNA532891	PRJNA125353	PRJNA613996
	PRJNA565327	PRJNA149901	PRJNA597692
	PRJNA353326	PRJNA270496	PRJNA599254
	PRJNA565979	PRJNA269153	PRJNA687397

	PRJNA498804	PRJNA313114	PRJNA577558
	PRJNA511456	PRJEB7860	PRJNA326133
	PRJNA412794	PRJNA794061	PRJNA358451
	PRJNA531411	PRJNA633502	PRJNA578485
	PRJNA531961	PRJNA715855	PRJNA475801
	PRJNA470932	PRJNA917827	PRJNA275817
	PRJNA540473	PRJNA832965	PRJNA285203
	PRJNA542257	PRJNA451252	PRJNA578679
	PRJNA545101	PRJNA622800	PRJNA264430
	PRJNA548009	PRJNA636017	PRJNA483000
	PRJNA551290	PRJNA636124	PRJNA524383
	PRJNA553210	PRJNA669818	PRJNA205988
	PRJNA560385	PRJNA716602	PRJNA209873
	PRJNA564101	PRJNA729223	PRJNA212573
	PRJNA573901	PRJNA739384	PRJNA221229
	PRJNA577632	PRJNA741886	PRJNA224307
	PRJNA587094	PRJNA807106	PRJNA225618
	PRJNA587527	PRJNA892719	PRJNA226087
	PRJNA593768	PRJNA820057	PRJNA231861
	PRJNA590028	PRJNA907407	PRJNA235193
	PRJNA552541	PRJNA884754	PRJNA244630
	PRJNA531443	PRJNA886327	PRJNA253118
	PRJNA559237	PRJNA900290	PRJNA257307
	PRJNA592333	PRJNA901060	PRJNA258581
	PRJNA487658	PRJNA904124	PRJNA259760
	PRJNA544700	PRJNA910965	PRJNA275630
	PRJNA557370	PRJNA916761	PRJNA277206
	PRJNA522677	PRJNA1001163	PRJNA280500
	PRJNA593170	PRJNA720387	PRJNA280610
	PRJNA513291	PRJNA380736	PRJNA283489
	PRJNA515942	PRJNA764736	PRJNA286306
	PRJNA516115	PRJNA910299	PRJNA270769
	PRJNA270582	PRJEB3209	PRJNA295087
	PRJNA276711	PRJNA900229	PRJNA296828
	PRJNA361295	PRJNA657466	PRJNA297201
	PRJNA380626	PRJNA668665	PRJNA298571
	PRJNA383542	PRJNA612602	PRJNA303041
	PRJNA515700	PRJNA863717	PRJNA304605
	PRJNA522353	PRJNA812897	PRJNA307573
	PRJNA497753	PRJNA728663	PRJNA322323
	PRJNA523227	PRJNA675767	PRJNA349805
	PRJNA514369	PRJNA824995	PRJNA352353
	PRJNA445391	PRJNA601637	PRJNA353659
	PRJNA451045	PRJNA775838	PRJNA368713
	PRJNA454461	PRJNA724674	PRJNA382193
	PRJNA496245	PRJNA684443	PRJNA382974
	PRJNA473287	PRJNA748771	PRJNA384391

	PRJNA471163	PRJNA675728	PRJNA391469
	PRJNA498760	PRJNA625995	PRJNA395067
	PRJNA479546	PRJNA728755	PRJNA397840
	PRJNA430730	PRJNA792029	PRJNA419825
	PRJNA381421	PRJNA713866	PRJNA427530
	PRJNA464393	PRJNA755472	PRJNA433362
	PRJNA472878	PRJNA833661	PRJNA435673
	PRJNA473503	PRJNA785744	PRJNA436581
	PRJNA476330	PRJNA689427	PRJNA445860
	PRJNA477827	PRJNA613361	PRJNA481472
	PRJNA355884	PRJNA916653	PRJNA483416
	PRJNA481032	PRJNA541564	PRJNA486660
	PRJNA488087	PRJNA589980	PRJNA489205
	PRJNA488612	PRJNA596181	PRJNA493813
	PRJNA489754	PRJNA603382	PRJNA497061
	PRJNA491711	PRJNA605197	PRJNA501897
	PRJNA494162	PRJNA608852	PRJNA501893
	PRJNA495373	PRJNA611997	PRJNA501912
	PRJNA497283	PRJNA613403	PRJNA509421
	PRJNA497288	PRJNA616363	PRJNA512101
	PRJNA355862	PRJNA623382	PRJNA512883
	PRJNA515491	PRJEB30255	PRJNA319489
	PRJNA518761	PRJEB30261	PRJNA321192
	PRJNA529472	PRJEB43322	PRJNA326510
	PRJNA534075	PRJEB43323	PRJNA330689
	PRJNA534076	PRJEB44081	PRJNA331206
	PRJNA547803	PRJEB44238	PRJNA335626
	PRJNA551302	PRJEB44716	PRJNA310615
	PRJNA554298	PRJEB46241	PRJNA339688
	PRJNA555854	PRJNA922529	PRJNA344999
	PRJNA578731	PRJNA896578	PRJNA348371
	PRJNA579592	PRJNA817609	PRJNA355266
	PRJNA137849	PRJNA901196	PRJNA358055
	PRJNA63447	PRJNA826542	PRJNA360533
	PRJNA151657	PRJNA809230	PRJNA358686
	PRJNA170436	PRJNA901619	PRJNA360952
	PRJNA171165	PRJNA972714	PRJNA361294
	PRJNA266892	PRJNA543865	PRJNA361474
	PRJNA266751	PRJNA602876	PRJNA361473
	PRJNA291089	PRJNA603291	PRJNA376728
	PRJNA530879	PRJNA603461	PRJNA377167
	PRJNA341815	PRJNA604769	PRJNA377169
	PRJNA268374	PRJNA609703	PRJNA377170
	PRJNA435442	PRJNA609717	PRJNA377713
	PRJEB65224	PRJNA610530	PRJNA328590
	PRJEB23952	PRJNA613736	PRJNA381596
	PRJEB22684	PRJEB36921	PRJNA385514

	PRJEB2908	PRJNA623501	PRJNA385567
	PRJEB4205	PRJNA623611	PRJNA386460
	PRJEB4744	PRJNA623615	PRJNA388401
	PRJEB6120	PRJNA623983	PRJNA389574
	PRJEB7389	PRJNA625054	PRJNA389727
	PRJEB26090	PRJNA624911	PRJNA392290
	PRJNA433666	PRJNA625655	PRJNA392924
	PRJNA547816	PRJNA625690	PRJNA393630
	PRJNA548337	PRJNA627134	PRJNA395735
	PRJNA355164	PRJNA533326	PRJNA396456
	PRJNA397747	PRJNA627881	PRJNA397844
	PRJNA704620	PRJNA628878	PRJNA398585
	PRJNA667898	PRJNA482135	PRJNA401809
	PRJNA722241	PRJNA630416	PRJNA406732
	PRJNA730915	PRJNA632042	PRJNA414057
	PRJNA612419	PRJNA633874	PRJNA417050
	PRJNA417267	PRJNA635600	PRJNA418636
	PRJNA213667	PRJNA635794	PRJNA418904
	PRJNA973039	PRJNA554684	PRJNA419209
	PRJNA877223	PRJNA637504	PRJNA420695
	PRJNA974439	PRJNA638018	PRJNA420970
	PRJNA978429	PRJNA638467	PRJNA422807
	PRJNA992479	PRJNA639721	PRJNA423015
	PRJNA998265	PRJNA639839	PRJNA427882
	PRJNA996215	PRJNA640046	PRJNA429035
	PRJNA976288	PRJNA640116	PRJNA433264
	PRJNA342202	PRJNA640142	PRJNA437287
	PRJNA757902	PRJNA640683	PRJNA438083
	PRJNA918720	PRJNA641366	PRJNA438276
	PRJNA790034	PRJNA600433	PRJNA438688
	PRJNA936010	PRJNA641624	PRJNA439246
	PRJNA775856	PRJNA641625	PRJNA448601
	PRJNA749882	PRJNA641623	PRJNA450408
	PRJNA856413	PRJNA645309	PRJNA453692
	PRJNA937865	PRJNA648763	PRJNA453915
	PRJNA793619	PRJNA649938	PRJNA454268
	PRJNA475674	PRJNA655121	PRJNA454386
	PRJNA750145	PRJNA655419	PRJNA454863
	PRJNA750397	PRJNA655456	PRJNA471827
	PRJNA750724	PRJNA655835	PRJNA471963
	PRJNA750731	PRJNA656214	PRJNA471962
	PRJNA752779	PRJNA657623	PRJNA472608
	PRJNA754125	PRJNA658496	PRJNA474443
	PRJNA754430	PRJNA659037	PRJNA475417
	PRJNA756193	PRJNA659038	PRJNA476786
	PRJNA757003	PRJNA659039	PRJNA476937
	PRJNA757015	PRJNA659731	PRJNA479873

	PRJNA757441	PRJNA662248	PRJNA481649
	PRJNA675015	PRJNA660634	PRJNA482134
	PRJNA757717	PRJNA663788	PRJNA482136
	PRJNA758046	PRJNA664367	PRJNA484941
	PRJNA758063	PRJNA664584	PRJNA492247
	PRJNA760461	PRJNA665035	PRJNA493621
	PRJNA760927	PRJNA665581	PRJNA497065
	PRJNA764910	PRJNA665692	PRJNA497814
	PRJNA765021	PRJNA666201	PRJNA445453
	PRJNA767388	PRJNA666670	PRJNA503400
	PRJNA694068	PRJNA666932	PRJNA504421
	PRJNA769091	PRJNA667921	PRJNA505814
	PRJNA769420	PRJNA668329	PRJNA505883
	PRJNA770616	PRJNA605114	PRJNA505884
	PRJNA770660	PRJNA671680	PRJNA506725
	PRJNA770771	PRJNA671682	PRJNA506986
	PRJNA773722	PRJNA671689	PRJNA507751
	PRJNA774056	PRJNA671693	PRJNA508892
	PRJNA774189	PRJNA673825	PRJNA509424
	PRJNA775872	PRJNA674644	PRJNA510079
	PRJNA776288	PRJNA674782	PRJNA512252
	PRJNA780304	PRJNA677887	PRJNA512491
	PRJNA782655	PRJNA679841	PRJNA513305
	PRJNA782745	PRJNA680481	PRJNA515538
	PRJNA783903	PRJNA680949	PRJNA515628
	PRJNA784969	PRJEB35440	PRJNA521496
	PRJNA785005	PRJNA690629	PRJNA522371
	PRJNA785830	PRJNA693459	PRJNA524346
	PRJNA786583	PRJNA700411	PRJNA526036
	PRJNA786617	PRJNA700720	PRJNA528188
	PRJNA786884	PRJNA701020	PRJNA528321
	PRJNA727579	PRJNA540988	PRJNA529694
	PRJNA793004	PRJNA701744	PRJNA532392
	PRJNA737801	PRJNA701894	PRJNA533594
	PRJNA797094	PRJNA701895	PRJNA533780
	PRJNA797428	PRJNA702068	PRJNA534479
	PRJNA798624	PRJNA704262	PRJNA540963
	PRJNA800759	PRJNA704264	PRJNA541296
	PRJNA553346	PRJNA704689	PRJNA541324
	PRJNA800802	PRJEB39794	PRJNA542907
	PRJNA800801	PRJNA715450	PRJNA543318
	PRJNA801289	PRJNA715920	PRJNA548813
	PRJNA681401	PRJNA716642	PRJNA550985
	PRJNA801683	PRJNA720037	PRJNA551745
	PRJNA802825	PRJNA720057	PRJNA551746
	PRJNA802849	PRJNA722728	PRJNA551747
	PRJNA802910	PRJNA723320	PRJNA551750

	PRJNA804551	PRJNA688630	PRJNA551749
	PRJNA804865	PRJNA723762	PRJNA551752
	PRJNA805011	PRJNA724027	PRJNA551751
	PRJNA807694	PRJNA724030	PRJNA551753
	PRJNA810490	PRJNA647916	PRJNA551754
	PRJNA813529	PRJNA727674	PRJNA551755
	PRJNA813860	PRJNA729461	PRJNA551756
	PRJNA814286	PRJNA729793	PRJNA551757
	PRJNA815310	PRJNA731473	PRJNA551758
	PRJNA817567	PRJNA727525	PRJNA551759
	PRJNA818624	PRJNA732920	PRJNA552334
	PRJNA824502	PRJNA634388	PRJNA556026
	PRJNA745097	PRJNA736741	PRJNA556809
	PRJNA826336	PRJNA738336	PRJNA556886
	PRJNA826335	PRJNA738841	PRJNA557038
	PRJNA827853	PRJEB34993	PRJNA557224
	PRJNA831873	PRJNA742116	PRJNA517490
	PRJNA832158	PRJNA743007	PRJNA562914
	PRJNA832150	PRJNA743678	PRJNA566010
	PRJNA836439	PRJNA745343	PRJNA566009
	PRJNA836498	PRJNA745470	PRJNA566149
	PRJNA837419	PRJNA747271	PRJNA576649
	PRJNA837585	PRJNA748741	PRJNA576650
	PRJNA837608	PRJNA749464	PRJNA576651
	PRJNA840916	PRJNA749814	PRJNA576652
	PRJNA841224	PRJNA686380	PRJNA577128
	PRJNA841670	PRJNA591214	PRJNA578565
	PRJNA717743	PRJNA492821	PRJNA578614
	PRJNA842417	PRJNA521800	PRJNA580238
	PRJNA842956	PRJNA389469	PRJNA580266
	PRJNA843034	PRJNA187456	PRJNA586855
	PRJNA846294	PRJNA203652	PRJNA587707
	PRJNA846773	PRJNA205165	PRJNA588963
	PRJNA849575	PRJNA213130	PRJNA590968
	PRJNA851969	PRJNA213629	PRJNA593230
	PRJNA853306	PRJNA215056	PRJNA593571
	PRJNA854896	PRJNA230112	PRJNA593727
	PRJNA858681	PRJNA231896	PRJNA595867
	PRJNA860213	PRJNA238879	PRJNA125509
	PRJNA860553	PRJNA242201	PRJNA145181
	PRJNA861984	PRJNA245235	PRJNA169928
	PRJNA864749	PRJNA246200	PRJNA541863
	PRJNA866163	PRJNA248315	PRJNA544353
	PRJNA874572	PRJNA253099	PRJNA247362
	PRJNA879057	PRJNA254155	PRJNA408072
	PRJNA885130	PRJNA254363	PRJNA399635
	PRJNA887060	PRJNA254362	PRJNA305666

	PRJNA890327	PRJNA254361	PRJEB21898
	PRJNA890585	PRJNA255679	PRJEB23565
	PRJNA890586	PRJNA257379	PRJEB23569
	PRJNA892998	PRJNA258219	PRJEB23692
	PRJNA894195	PRJNA260073	PRJEB23752
	PRJNA895407	PRJNA261235	PRJEB23801
	PRJNA899846	PRJNA261331	PRJEB25002
	PRJNA903902	PRJNA268578	PRJEB6651
	PRJNA905815	PRJNA274997	PRJEB27857
	PRJNA908728	PRJNA276134	PRJNA263979
	PRJNA908733	PRJNA277309	PRJNA419725
	PRJNA909819	PRJNA277845	PRJNA401377
	PRJNA911261	PRJNA281255	PRJNA559201
	PRJNA885440	PRJNA282486	PRJNA718097
	PRJNA914218	PRJNA283872	PRJDB11308
	PRJNA914726	PRJNA284147	PRJNA700773
	PRJNA922130	PRJNA285284	PRJNA793881
	PRJNA931610	PRJNA288467	PRJNA547697
	PRJNA931609	PRJNA288805	PRJNA1001316
	PRJNA933532	PRJNA289388	PRJNA556576
	PRJNA935340	PRJNA291872	PRJNA382244
	PRJNA935733	PRJNA294657	PRJNA564901
	PRJNA763228	PRJNA296819	PRJNA169329
	PRJNA687289	PRJNA296900	PRJNA910966
	PRJNA910343	PRJNA298926	PRJNA564561
	PRJNA884760	PRJNA299809	PRJNA938445
	PRJNA830998	PRJNA307274	PRJNA105719
	PRJNA954055	PRJNA308081	PRJNA721628
	PRJNA811343	PRJNA308305	PRJNA564396
	PRJNA871962	PRJNA312400	PRJNA287115
	PRJNA943413	PRJNA314523	PRJNA314248
	PRJEB50576	PRJNA316610	PRJNA833878
	PRJEB52408	PRJNA318165	PRJNA761712
	PRJNA776388	PRJNA309292	PRJNA602480
	PRJNA850444	PRJNA311970	PRJNA602696
	PRJNA657546	PRJNA314993	PRJNA604380
	PRJNA678596	PRJNA314998	PRJNA604999
	PRJNA661202	PRJNA315000	PRJNA605255
	PRJNA680213	PRJNA316214	PRJNA607300
	PRJNA855586	PRJNA316856	PRJNA607299
	PRJNA834597	PRJNA318051	PRJNA612488
	PRJNA429173	PRJNA321872	PRJNA612700
	PRJNA487532	PRJNA321870	PRJNA626578
	PRJNA541878	PRJNA322561	PRJNA627241
	PRJNA545730	PRJNA329255	PRJNA628681
	PRJNA547808	PRJNA330795	PRJNA629288
	PRJNA548806	PRJNA348110	PRJNA630977

	PRJNA549597	PRJNA350708	PRJNA631548
	PRJNA551384	PRJNA350801	PRJNA631957
	PRJNA553514	PRJNA350803	PRJNA631994
	PRJNA555415	PRJNA338759	PRJNA634876
	PRJNA556704	PRJNA356173	PRJNA648742
	PRJNA556650	PRJNA358481	PRJNA656286
	PRJNA556651	PRJNA359691	PRJNA657062
	PRJNA556652	PRJNA359690	PRJNA664207
	PRJNA556653	PRJNA360136	PRJNA671711
	PRJNA556654	PRJNA361293	PRJNA681212
	PRJNA557481	PRJNA369756	PRJNA681592
	PRJNA562384	PRJNA371822	PRJNA682997
	PRJNA562493	PRJNA374355	PRJNA682998
	PRJNA563562	PRJNA374551	PRJNA683140
	PRJNA563900	PRJNA376426	PRJNA683599
	PRJNA573631	PRJNA377912	PRJNA697861
	PRJNA574846	PRJNA369681	PRJNA698551
	PRJNA578532	PRJNA383562	PRJNA704408
	PRJNA590355	PRJNA388648	PRJNA706673
	PRJNA590718	PRJNA394695	PRJNA718239
	PRJNA591619	PRJNA397181	PRJNA721306
	PRJNA593498	PRJNA398679	PRJNA722749
	PRJNA594380	PRJNA406823	PRJNA726809
	PRJNA595401	PRJNA407376	PRJNA727096
	PRJNA598734	PRJNA407773	PRJNA727298
	PRJNA598737	PRJNA412850	PRJNA737561
	PRJNA600540	PRJNA414779	PRJNA744531
	PRJNA600880	PRJNA415030	PRJNA749560
	PRJNA600884	PRJNA415032	PRJNA755604
	PRJNA605248	PRJNA415031	PRJNA759272
	PRJNA605796	PRJNA415033	PRJNA763796
	PRJNA606492	PRJNA415034	PRJNA769094
	PRJNA607197	PRJNA415171	PRJNA772277
	PRJNA607231	PRJNA416702	PRJNA776879
	PRJNA607999	PRJNA430072	PRJNA778709
	PRJNA608297	PRJNA433561	PRJNA780821
	PRJNA615233	PRJNA434658	PRJNA784305
	PRJNA625487	PRJNA445182	PRJNA785255
	PRJNA625891	PRJNA451492	PRJNA786346
	PRJNA627155	PRJNA453130	PRJNA787288
	PRJNA630233	PRJNA453384	PRJNA790822
	PRJNA634100	PRJNA453422	PRJNA803212
	PRJNA637897	PRJNA453536	PRJNA803469
	PRJNA644639	PRJNA472627	PRJNA816153
	PRJNA645937	PRJNA472770	PRJNA819404
	PRJNA646706	PRJNA473979	PRJNA820713
	PRJNA648161	PRJNA475639	PRJNA820719

	PRJNA661188	PRJNA476121	PRJNA824878
	PRJNA662582	PRJNA476616	PRJNA826446
	PRJNA664116	PRJNA481471	PRJNA826476
	PRJNA665988	PRJNA483400	PRJNA826484
	PRJNA667952	PRJNA484192	PRJNA830860
	PRJNA681559	PRJNA485413	PRJNA830904
	PRJNA605252	PRJNA450599	PRJNA832764
	PRJNA695223	PRJNA497062	PRJNA837211
	PRJNA695562	PRJNA497256	PRJNA859779
	PRJNA702160	PRJNA498321	PRJNA861993
	PRJNA703841	PRJNA498517	PRJNA866129
	PRJNA706409	PRJNA498519	PRJNA881331
	PRJNA718086	PRJNA498527	PRJNA883942
	PRJNA718085	PRJNA498526	PRJNA897861
	PRJNA725118	PRJNA498529	PRJNA897868
	PRJNA725794	PRJNA501896	PRJNA902287
	PRJNA731102	PRJNA505780	PRJNA907376
	PRJNA731488	PRJNA509149	PRJNA913131
	PRJNA734658	PRJNA515042	PRJNA916473
	PRJNA736259	PRJNA515486	PRJNA925562
	PRJNA736612	PRJNA518162	PRJNA928485
	PRJNA745989	PRJNA524475	PRJNA930839
	PRJNA746479	PRJNA526909	PRJNA935379
	PRJNA757275	PRJNA487078	PRJNA938751
	PRJNA766610	PRJNA533133	PRJNA947064
	PRJNA767378	PRJNA533132	PRJNA947663
	PRJNA777275	PRJNA534074	PRJNA978055
	PRJNA779733	PRJNA136221	PRJNA985774
	PRJNA811580	PRJNA153959	PRJNA946546
	PRJNA627652	PRJNA153961	PRJNA870964
	PRJNA826893	PRJNA145711	PRJNA607816
	PRJNA846161	PRJNA147397	PRJNA726947
	PRJNA781565	PRJNA151813	PRJNA881928
	PRJNA847236	PRJNA157515	PRJNA801931
	PRJNA856248	PRJNA157987	PRJNA753061
	PRJNA869065	PRJNA159827	PRJNA609330
	PRJNA895374	PRJNA161009	PRJNA1001149
	PRJNA897891	PRJNA162879	PRJNA724704
	PRJNA915973	PRJNA163111	PRJNA923732
	PRJNA916903	PRJNA167851	PRJEB48601
	PRJNA954018	PRJNA167443	PRJEB55316
	PRJNA961125	PRJNA167768	PRJEB55398
	PRJNA975594	PRJNA170090	PRJEB52166
	PRJNA975833	PRJNA171939	PRJEB52610
	PRJNA692831	PRJNA172917	PRJEB33509
	PRJNA831775	PRJNA173462	PRJEB41085
	PRJNA846274	PRJNA239705	PRJEB41890

	PRJNA566441	PRJNA369552	PRJEB46065
	PRJNA685783	PRJNA421072	PRJNA782183
	PRJNA603843	PRJNA386575	PRJNA771685
	PRJNA824272	PRJNA257532	PRJNA797913
	PRJNA965937	PRJNA316524	PRJNA563539
	PRJNA816530	PRJNA341059	PRJNA481726
	PRJNA641646	PRJNA412440	PRJNA481648
	PRJNA853906	PRJNA171327	PRJNA306694
	PRJNA613350	PRJNA484547	PRJNA374033
	PRJNA643769	PRJNA245463	PRJNA336351
	PRJDB9451	PRJNA400256	PRJNA384390
	PRJDB12380	PRJNA396095	PRJNA348736
	PRJEB47673	PRJNA301134	PRJNA395030
	PRJEB53738	PRJNA246023	PRJNA399094
	PRJEB43689	PRJNA241095	PRJNA415408
	PRJEB57225	PRJDB1099	PRJNA419212
	PRJEB57800	PRJDA34559	PRJNA419824
	PRJEB51554	PRJEB19678	PRJNA431313
	PRJEB29402	PRJEB20138	PRJNA438473
	PRJEB32284	PRJEB65039	PRJNA476057
	PRJEB32349	PRJEB24882	PRJNA477931
	PRJEB33667	PRJEB4749	PRJNA482858
	PRJEB34179	PRJEB6115	PRJNA484982
	PRJEB36018	PRJEB6351	PRJNA435992
	PRJEB44738	PRJEB7282	PRJNA493099
	PRJEB47097	PRJEB7297	PRJNA505065
	PRJNA827119	PRJEB7300	PRJNA505172
	PRJNA787019	PRJEB7858	PRJNA511908
	PRJNA714135	PRJEB11541	PRJNA513055
	PRJNA352073	PRJEB11549	PRJNA522460
	PRJNA415678	PRJEB11558	PRJNA522462
	PRJDB3120	PRJEB11957	PRJNA541584
	PRJDB3776	PRJEB14143	PRJNA547807
	PRJNA310826	PRJEB27318	PRJNA547814
	PRJNA181016	PRJEB31662	PRJNA549932
	PRJNA533136	PRJEB19388	PRJNA552685
	PRJNA175949	PRJNA475188	PRJNA556615
	PRJNA176460	PRJNA431389	PRJNA557353
	PRJNA184836	PRJNA471349	PRJNA558168
	PRJNA186746	PRJNA254500	PRJNA562385
	PRJNA186963	PRJNA305981	PRJNA562527
	PRJNA189717	PRJNA337889	PRJNA594409
	PRJNA190655	PRJNA219215	PRJNA598199
	PRJNA203013	PRJNA668112	PRJNA600922
	PRJNA203205	PRJNA685412	PRJNA445481
	PRJNA203446	PRJNA689606	PRJNA483305
	PRJNA203678	PRJNA688837	PRJNA481798

	PRJNA209374	PRJEB40872	PRJEB23988
	PRJNA211869	PRJNA713314	PRJEB25111
	PRJNA219483	PRJNA660882	PRJEB14790
	PRJNA229103	PRJDB8471	PRJNA516147
	PRJNA229376	PRJEB44348	PRJNA359682
	PRJNA229375	PRJDB8730	PRJNA315675
	PRJNA215685	PRJNA791133	PRJNA193306
	PRJNA230574	PRJNA916520	PRJNA239327
	PRJNA230611	PRJNA634718	PRJNA275667
	PRJNA230643	PRJNA659224	PRJNA289568
	PRJNA239324	PRJDB5492	PRJNA297633
	PRJNA239339	PRJNA238349	PRJNA299151
	PRJNA244434	PRJNA258538	PRJNA299280
	PRJNA244784	PRJNA301943	PRJNA312116
	PRJNA246451	PRJNA400164	PRJNA324414
	PRJNA255768	PRJNA400597	PRJNA324679
	PRJNA256175	PRJNA476300	PRJNA327415
	PRJNA260289	PRJNA524799	PRJNA327927
	PRJNA262085	PRJNA573100	PRJNA336176
	PRJNA263394	PRJNA608096	PRJNA336449
	PRJNA266231	PRJNA622540	PRJNA343830
	PRJNA267836	PRJNA625752	PRJNA351228
	PRJNA267838	PRJNA655251	PRJNA834813
	PRJNA268010	PRJNA655413	PRJNA904326
	PRJNA268365	PRJNA162885	PRJNA278702
	PRJNA268974	PRJNA141085	PRJNA266072
	PRJNA275629	PRJNA170184	PRJNA260556
	PRJNA275670	PRJNA509912	PRJNA171282
	PRJNA275863	PRJDB5704	PRJNA254534
	PRJNA275975	PRJDB9068	PRJEB11818
	PRJNA277042	PRJNA309297	PRJEB10234
	PRJNA283807	PRJNA522469	PRJNA344790
	PRJNA283266	PRJNA703962	PRJNA698585
	PRJNA283345	PRJEB11647	PRJEB62424
	PRJNA284262	PRJNA322534	PRJEB64882
	PRJNA284738	PRJNA322528	PRJNA874842
	PRJNA289566	PRJNA245418	PRJNA601752
	PRJNA289569	PRJNA645715	PRJNA307572
	PRJNA290333	PRJNA763395	PRJNA308199
	PRJNA294738	PRJNA692075	PRJNA474911
	PRJNA296059	PRJNA611889	PRJNA308200
	PRJNA302653	PRJNA612416	
European Nucleotide Archive	PRJNA996921	PRJNA808880	PRJNA381759
	PRJEB55317	PRJNA808972	PRJNA398048
	PRJNA801595	PRJNA813850	PRJNA434583
	PRJNA892960	PRJNA814517	PRJNA454668
	PRJNA733683	PRJNA835141	PRJNA454683

	PRJNA833124	PRJNA839264	PRJNA472877
	PRJNA847957	PRJNA847974	PRJNA498063
	PRJNA796359	PRJNA872361	PRJNA498065
	PRJNA757179	PRJNA887334	PRJNA498064
	PRJNA766437	PRJNA893546	PRJNA504423
	PRJNA753105	PRJNA901016	PRJNA522238
	PRJNA684608	PRJNA902380	PRJNA523191
	PRJNA595157	PRJNA902382	PRJNA526737
	PRJNA625666	PRJNA902384	PRJNA530210
	PRJNA657833	PRJNA914301	PRJNA532933
	PRJNA661464	PRJNA915472	PRJNA533094
	PRJNA574182	PRJNA925867	PRJNA533109
	PRJNA553214	PRJNA937216	PRJNA553212
	PRJNA515359	PRJNA939119	PRJNA553567
	PRJNA357200	PRJNA750575	PRJNA559139
	PRJNA300429	PRJNA879104	PRJNA565401
	PRJNA185008	PRJNA731018	PRJNA282167
	PRJNA244586	PRJNA728962	PRJNA305831
	PRJNA189267	PRJNA956723	PRJNA255833
	PRJNA730314	PRJNA856069	PRJNA416676
	PRJNA518134	PRJNA818975	PRJNA592597
	PRJNA531840	PRJNA905219	PRJNA495615
	PRJNA605553	PRJEB54976	PRJEB19964
	PRJNA941970	PRJNA846525	PRJEB20597
	PRJNA565658	PRJNA866532	PRJEB23544
	PRJNA615220	PRJNA879724	PRJNA505781
	PRJNA605353	PRJNA598586	PRJNA520804
	PRJNA474484	PRJNA599328	PRJNA503657
	PRJNA725266	PRJNA623612	PRJNA858488
	PRJNA775974	PRJNA623610	PRJNA837885
	PRJNA790998	PRJNA625654	PRJNA872200
	PRJNA854387	PRJNA543237	PRJNA831808
	PRJNA390793	PRJNA635732	PRJNA716338
	PRJNA486160	PRJNA648960	PRJNA591625
	PRJNA497122	PRJNA663360	PRJNA257386
	PRJNA393765	PRJNA664055	PRJNA764724
	PRJNA298782	PRJNA660112	PRJNA659255
	PRJDB2619	PRJNA679255	PRJNA615805
	PRJNA322082	PRJNA680488	PRJNA189716
	PRJNA722240	PRJNA685790	PRJNA379938
	PRJNA344715	PRJNA702589	PRJNA390794
	PRJNA345161	PRJNA718205	PRJNA550113
	PRJNA977999	PRJNA722575	PRJNA776158
	PRJNA984179	PRJNA731456	PRJNA764207
	PRJNA994488	PRJEB43339	PRJNA601934
	PRJNA773257	PRJNA736485	PRJNA704548
	PRJNA794937	PRJNA737758	PRJNA698573

	PRJNA912644	PRJNA744781	PRJNA382988
	PRJNA769430	PRJNA476767	PRJNA382987
	PRJNA772934	PRJNA386593	PRJNA382986
	PRJNA774812	PRJNA559230	PRJNA382984
	PRJNA778072	PRJNA240075	PRJNA488615
	PRJNA800803	PRJNA298568	PRJNA422806
	PRJNA803151	PRJNA356287	PRJNA275262
	PRJNA803200	PRJNA381134	PRJNA522989
	PRJNA807506	PRJNA381599	PRJNA556491
	PRJNA343110	PRJNA658989	
iProX	IPX0001393000	IPX0003143000	IPX0005091000
	IPX0001566000	IPX0003148000	IPX0005219000
	IPX0001899000	IPX0003450000	IPX0005343000
	IPX0002190000	IPX0003537000	IPX0005406000
	IPX0002409000	IPX0004049000	IPX0005554000
	IPX0002485000	IPX0004094000	IPX0005567000
	IPX0002495000	IPX0004099000	IPX0005679000
	IPX0002585000	IPX0004252000	IPX0005829000
	IPX0002586000	IPX0004368000	IPX0005942000
	IPX0002650000	IPX0004455000	IPX0005953000
	IPX0003136000	IPX0004707000	IPX0006153000
	IPX0003138000	IPX0004941000	IPX0006271000
	IPX0003142000	IPX0005036000	IPX0006583000
	IPX0006825000		
jPOST	JPST000036	JPST000914	JPST001372
	JPST000115	JPST000918	JPST001374
	JPST000386	JPST000925	JPST001390
	JPST000398	JPST000926	JPST001400
	JPST000422	JPST000931	JPST001478
	JPST000450	JPST000952	JPST001502
	JPST000491	JPST000989	JPST001505
	JPST000527	JPST000990	JPST001513
	JPST000528	JPST001007	JPST001529
	JPST000529	JPST001036	JPST001533
	JPST000531	JPST001132	JPST001536
	JPST000571	JPST001151	JPST001574
	JPST000591	JPST001186	JPST001580
	JPST000617	JPST001188	JPST001604
	JPST000618	JPST001223	JPST001652
	JPST000644	JPST001242	JPST001682
	JPST000675	JPST001262	JPST001708
	JPST000700	JPST001266	JPST001709
	JPST000763	JPST001267	JPST001710
	JPST000786	JPST001278	JPST001781
	JPST000828	JPST001304	JPST001794
	JPST000829	JPST001331	JPST001805
	JPST000872	JPST001358	JPST001816

	JPST000875	JPST001359	JPST001923
	JPST000911	JPST001360	JPST001939
	JPST001942	JPST002184	
MassIVE	MSV000078532	MSV000078532	MSV000086569
	MSV000078609	MSV000078609	MSV000086613
	MSV000078997	MSV000078997	MSV000086667
	MSV000078998	MSV000078998	MSV000086668
	MSV000078999	MSV000078999	MSV000086669
	MSV000079000	MSV000079000	MSV000086671
	MSV000079001	MSV000079001	MSV000086672
	MSV000079002	MSV000079002	MSV000086769
	MSV000079003	MSV000079003	MSV000086855
	MSV000079004	MSV000079004	MSV000087036
	MSV000079005	MSV000079005	MSV000087415
	MSV000079006	MSV000079006	MSV000087416
	MSV000079007	MSV000079007	MSV000087417
	MSV000079008	MSV000079008	MSV000087418
	MSV000079009	MSV000079009	MSV000087587
	MSV000079010	MSV000079010	MSV000087693
	MSV000079082	MSV000079082	MSV000087700
	MSV000079100	MSV000079100	MSV000087850
	MSV000079567	MSV000079567	MSV000087994
	MSV000079669	MSV000079669	MSV000088220
	MSV000079722	MSV000079722	MSV000088402
	MSV000079841	MSV000079841	MSV000088577
	MSV000080175	MSV000080175	MSV000088581
	MSV000080298	MSV000080298	MSV000088631
	MSV000080631	MSV000080631	MSV000088652
	MSV000080690	MSV000080690	MSV000088653
	MSV000080703	MSV000080703	MSV000088822
	MSV000080733	MSV000080733	MSV000088866
	MSV000080759	MSV000080759	MSV000089185
	MSV000080772	MSV000080772	MSV000089252
	MSV000080807	MSV000080807	MSV000089341
	MSV000080808	MSV000080808	MSV000089357
	MSV000081009	MSV000081009	MSV000089362
	MSV000081355	MSV000081355	MSV000089457
	MSV000081356	MSV000081356	MSV000089463
	MSV000081360	MSV000081360	MSV000089597
	MSV000081361	MSV000081361	MSV000090044
	MSV000081363	MSV000081363	MSV000090045
	MSV000081411	MSV000081411	MSV000090175
	MSV000081412	MSV000081412	MSV000090492
	MSV000081413	MSV000081413	MSV000090595
	MSV000081414	MSV000081414	MSV000090795
	MSV000081439	MSV000081439	MSV000090867
	MSV000081483	MSV000081483	MSV000090908

	MSV000081663	MSV000081663	MSV000090932
	MSV000081926	MSV000081926	MSV000091397
	MSV000081947	MSV000081947	MSV000091458
	MSV000081948	MSV000081948	MSV000091646
	MSV000081962	MSV000081962	MSV000091669
	MSV000082079	MSV000082079	MSV000091836
	MSV000082154	MSV000082154	MSV000091953
PRIDE	PXD000357	PXD030086	PXD015466
	PXD000384	PXD025794	PXD027487
	PXD000415	PXD029303	PXD024120
	PXD000680	PXD027126	PXD005878
	PXD000705	PXD019664	PXD028150
	PXD000883	PXD028186	PXD031140
	PXD000933	PXD021225	PXD012549
	PXD001281	RPXD034664	PXD020806
	PXD001434	RPXD034675	PXD031261
	PXD001711	RPXD034676	PXD009523
	PXD002058	RPXD034677	PXD030415
	PXD002133	PXD013614	PXD035996
	PXD002322	PXD028756	PXD011322
	PXD002383	PXD026284	PXD004286
	PXD002389	PXD027473	PXD031702
	PXD002395	PXD034231	PXD033958
	PXD002412	PXD009147	PXD007049
	PXD002534	PXD025038	PXD033939
	PXD002601	PXD014901	PXD014733
	PXD002613	PXD026813	PXD018346
	PXD002676	PXD010551	PXD020431
	PXD002733	PXD027400	PXD033067
	PXD003053	PXD022476	PXD021039
	PXD003278	PXD008890	PXD030970
	PXD003310	PXD027472	PXD018742
	PXD003431	PXD033731	PXD031678
	PXD003509	PXD020658	PXD015008
	PXD003700	PXD034227	PXD015208
	PXD004026	PXD021257	PXD033651
	PXD004288	PXD032881	PXD033629
	PXD004358	PXD001468	PXD033630
	PXD004494	PXD009547	PXD033631
	PXD004595	PXD013348	PXD015884
	PXD004659	PXD006094	PXD013685
	PXD004668	PXD033572	PXD011786
	PXD004884	PXD040741	PXD011879
	PXD004886	PXD005097	PXD022279
	PXD005086	PXD016934	PXD027477
	PXD005120	PXD001956	PXD031319
	PXD005218	PXD015528	PXD021942

	PXD005542	PXD015531	PXD005583
	PXD005567	PXD015530	PXD023479
	PXD005573	PXD015554	PXD018902
	PXD005584	PXD038864	PXD009731
	PXD005633	PXD023462	PXD023136
	PXD005761	PXD023463	PXD010796
	PXD006391	PXD023461	PXD031114
	PXD006514	PXD023464	PXD014678
	PXD006690	PXD023465	PXD030304
	PXD007038	PXD027983	PXD037154
	PXD007629	PXD001863	PXD011727
	PXD007991	PXD017598	PXD028541
	PXD008498	PXD029786	PXD011124
	PXD008509	PXD011698	PXD013097
	PXD009252	PXD015918	PXD015845
	PXD009267	PXD007554	PXD010908
	PXD009292	PXD037779	PXD012713
	PXD009335	PXD030475	PXD014199
	PXD009803	PXD042246	PXD025725
	PXD009867	PXD020533	PXD003784
	PXD009966	PXD006221	PXD037866
	PXD010520	PXD006220	PXD026071
	PXD010595	PXD006206	PXD003795
	PXD010788	PXD005233	PXD032138
	PXD011033	PXD022041	PXD009366
	PXD011093	PXD008997	PXD011756
	PXD011108	PXD044418	PXD012277
	PXD011173	PXD015860	PXD037572
	PXD011252	PXD030490	PXD022178
	PXD011369	PXD035651	PXD015316
	PXD011525	PXD035642	PXD021904
	PXD011881	PXD010914	PXD036424
	PXD012581	PXD003514	PXD033693
	PXD012669	PXD000343	PXD037694
	PXD012759	PXD016025	PXD017344
	PXD012924	PXD007199	PXD005857
	PXD012957	PXD007200	PXD031543
	PXD013085	PXD007201	PXD008365
	PXD013328	PXD036233	PXD026545
	PXD013332	PXD000026	PXD036040
	PXD013461	PXD003279	PXD004518
	PXD013779	PXD001696	PXD029642
	PXD014226	PXD001697	PXD031350
	PXD014534	PXD001698	PXD010144
	PXD014771	PXD001699	PXD005739
	PXD015243	PXD001700	PXD016041
	PXD015460	PXD001701	PXD031606

	PXD015485	PXD001706	PXD028397
	PXD015581	PXD001705	PXD011865
	PXD015963	PXD001707	PXD009830
	PXD015984	PXD001708	PXD038004
	PXD016166	PXD001709	PXD015779
	PXD016341	PXD001704	PXD015659
	PXD016417	PXD001702	PXD021217
	PXD016458	PXD001703	PXD012521
	PXD016532	PXD015972	PXD028935
	PXD016638	PXD011432	PXD006722
	PXD016681	PXD024157	PXD001672
	PXD016778	PXD010026	PXD013856
	PXD017030	PXD016371	PXD015912
	PXD017541	PXD018158	PXD019815
	PXD017646	PXD033208	PXD011840
	PXD017807	PXD013975	PXD024127
	PXD017852	PXD012197	PXD036741
	PXD017977	PXD026571	PXD015406
	PXD018114	PXD004000	PXD014820
	PXD018182	PXD012807	PXD036032
	PXD018517	PXD018125	PXD015075
	PXD018621	PXD009187	PXD015076
	PXD018894	PXD008117	PXD022111
	PXD018905	PXD008749	PXD032365
	PXD018976	PXD001308	PXD035503
	PXD018990	PXD017669	PXD002946
	PXD019137	PXD017685	PXD000932
	PXD019209	PXD017686	PXD012774
	PXD019337	PXD017664	PXD023906
	PXD019359	PXD017667	PXD015877
	PXD019417	PXD009908	PXD015789
	PXD019427	PXD032373	PXD025279
	PXD019702	PXD003831	PXD028701
	PXD019826	PXD003116	PXD027480
	PXD019848	PXD037678	PXD023957
	PXD019854	PXD015479	PXD027346
	PXD019878	PXD008963	PXD013636
	PXD020104	PXD017161	PXD013647
	PXD020231	PXD033959	PXD026647
	PXD020239	PXD034650	PXD030072
	PXD020305	PXD036624	PXD026793
	PXD020367	PXD033454	PXD015828
	PXD020600	PXD009028	PXD027479
	PXD020792	PXD013019	PXD034412
	PXD021040	PXD034847	PXD036995
	PXD021143	PXD008731	PXD016728
	PXD021320	PXD029091	PXD032721

	PXD021704	PXD034240	PXD045407
	PXD021768	PXD029034	PXD001609
	PXD021793	PXD012286	PXD023999
	PXD021808	PXD023548	PXD000685
	PXD022032	PXD033107	PXD030216
	PXD022091	PXD025829	PXD016314
	PXD022104	PXD018117	PXD027149
	PXD022589	PXD008349	PXD014524
	PXD022797	PXD027358	PXD027202
	PXD023025	PXD010688	PXD014473
	PXD023260	PXD030166	PXD001085
	PXD023367	PXD008318	PXD027138
	PXD023655	PXD008319	PXD027207
	PXD024052	PXD013508	PXD009178
	PXD024407	PXD010924	PXD009189
	PXD024524	PXD016007	PXD009051
	PXD024791	PXD012378	PXD032743
	PXD024851	PXD024350	PXD032836
	PXD025407	PXD021899	PXD032750
	PXD025443	PXD018002	PXD032849
	PXD025655	PXD037311	PXD032791
	PXD025763	PXD039085	PXD029456
	PXD026020	PXD032720	PXD016931
	PXD026292	PXD032718	PXD011269
	PXD026450	PXD027727	PXD032235
	PXD026657	PXD013116	PXD012174
	PXD026731	PXD018207	PXD003370
	PXD026747	PXD016613	PXD002646
	PXD026749	PXD016147	PXD005090
	PXD026750	PXD016766	PXD017713
	PXD026894	PXD022187	PXD013542
	PXD027394	PXD031863	PXD029644
	PXD027494	PXD026453	PXD020010
	PXD028149	PXD018979	PXD001511
	PXD028314	PXD026986	PXD033084
	PXD028404	PXD026740	PXD033671
	PXD029156	PXD006712	PXD036297
	PXD029429	PXD009866	PXD012077
	PXD029869	PXD001558	PXD010931
	PXD029885	PXD027257	PXD003133
	PXD030532	PXD030633	PXD003134
	PXD030548	PXD009786	PXD003896
	PXD031252	PXD006091	PXD029354
	PXD031276	PXD018294	PXD017774
	PXD031355	PXD020323	PXD028959
	PXD031374	PXD044625	PXD035227
	PXD031395	PXD014736	PXD010930

	PXD031408	PXD026830	PXD033451
	PXD031456	PXD031825	PXD017933
	PXD032095	PXD022017	PXD039259
	PXD032149	PXD028685	PXD003888
	PXD032155	PXD033960	PXD014661
	PXD032284	PXD016256	PXD033691
	PXD032716	PXD008343	PXD025661
	PXD033004	PXD037312	PXD017714
	PXD033233	PXD027234	PXD031482
	PXD033580	PXD027919	PXD023597
	PXD033780	PXD025817	PXD017673
	PXD033955	PXD012353	PXD037037
	PXD034115	PXD009865	PXD009095
	PXD034420	PXD009835	PXD014746
	PXD034833	PXD009851	PXD028769
	PXD034964	PXD009955	PXD002127
	PXD035142	PXD033716	PXD027300
	PXD035183	PXD015756	PXD028154
	PXD035184	PXD008400	PXD020630
	PXD035268	PXD008401	PXD000623
	PXD035774	PXD006633	PXD031644
	PXD035844	PXD031683	PXD001197
	PXD035846	PXD023221	PXD038921
	PXD037165	PXD002743	PXD023841
	PXD037383	PXD000277	PXD005955
	PXD037443	PXD012415	PXD023542
	PXD037472	PXD007761	PXD016149
	PXD040094	PXD023135	PXD034999
	PXD041156	PXD019066	PXD020165
	PXD039746	PXD014764	PXD018896
	PXD041361	PXD015077	PXD037630
	PXD019062	PXD021785	PXD004666
	PXD040319	PXD023984	PXD000197
	PXD032990	PXD011860	PXD038718
	PXD03868	PXD034396	PXD005482
	PXD044162	PXD019926	PXD035040
	PXD036924	PXD014549	PXD007584
	PXD037890	PXD012318	PXD007585
	PXD040301	PXD032740	PXD033764
	PXD016611	PXD032741	PXD031527
	PXD033926	PXD010108	PXD041792
	PXD021572	PXD009409	PXD013232
	PXD035373	PXD009861	PXD030285
	PXD041079	PXD034938	PXD037928
	PXD043036	PXD023139	PXD034557
	PXD039750	PXD020914	PXD004303
	PXD031655	PXD015155	PXD037929

	PXD032380	PXD008897	PXD009026
	PXD022536	PXD015978	PXD015104
	PXD036955	PXD020428	PXD024508
	PXD026511	PXD023943	PXD001958
	PXD023002	PXD021321	PXD024666
	PXD026520	PXD031515	PXD011835
	PXD026525	PXD022407	PXD013501
	PXD038125	PXD013192	PXD014909
	PXD021650	PXD017857	PXD002462
	PXD028195	PXD017855	PXD001383
	PXD029412	PXD017856	PXD003929
	PXD026519	PXD017858	PXD029508
	PXD026515	PXD017859	PXD041550
	PXD031303	PXD017851	PXD023527
	PXD026479	PXD017860	PXD036379
	PXD038683	PXD018252	PXD029504
	PXD022360	PXD017861	PXD024290
	PXD022341	PXD007845	PXD026523
	PXD014217	PXD029738	PXD024602
	PXD011941	PXD021712	PXD007570
	PXD027677	PXD030635	PXD039127
	PXD016409	PXD033498	PXD011584
	PXD033730	PXD041334	PXD033460
	PXD032379	PXD000593	PXD024387
	PXD014218	PXD017384	PXD037467
	PXD011946	PXD028246	PXD022346
	PXD011939	PXD023758	PXD028860
	PXD026295	PXD015693	PXD030601
	PXD036256	PXD004649	PXD039392
	PXD038445	PXD011728	PXD012350
	RPXD034322	PXD017109	PXD006362
	PXD020598	PXD029623	PXD014410
	PXD020599	PXD019428	PXD038474
	PXD038094	PXD028797	PXD026524
	PXD023208	PXD039467	PXD015830
	PXD013154	PXD008824	PXD027137
	PXD012021	PXD020090	PXD028864
	PXD020707	PXD035501	PXD010269
	PXD010350	PXD014533	PXD031311
	PXD018032	PXD023279	PXD016978
	PXD020307	PXD020018	PXD028148
	PXD032255	PXD018715	PXD035624
	PXD038562	PXD015454	PXD039071
	PXD029814	PXD024279	PXD006698
	PXD036054	PXD028110	PXD011126
	PXD042812	PXD004246	PXD029528
	PXD031917	PXD005643	PXD028982

	PXD031938	PXD013668	PXD038123
Metabolights	MTBLS61	MTBLS4268	MTBLS106
	MTBLS1358	MTBLS1014	
Metabolomics Workbench	PR001130		
